# The circadian dynamics of small nucleolar RNA in the mouse liver

**DOI:** 10.1098/rsif.2017.0034

**Published:** 2017-05-03

**Authors:** Stuart Aitken, Colin A. Semple

**Affiliations:** MRC Human Genetics Unit, Institute of Genetics and Molecular Medicine, University of Edinburgh, Edinburgh EH4 2XU, UK

**Keywords:** circadian rhythms, small nucleolar RNA, non-coding RNA, RNA dynamics

## Abstract

The circadian regulation of gene expression allows plants and animals to anticipate predictable environmental changes. While the influence of the circadian clock has recently been shown to extend to ribosome biogenesis, the dynamics and regulation of the many small nucleolar RNA that are required in pre-ribosomal RNA folding and modification are unknown. Using a novel computational method, we show that 18S and 28S pre-rRNA are subject to circadian regulation in a nuclear RNA sequencing time course. A population of snoRNA with circadian expression is identified that is functionally associated with rRNA modification. More generally, we find the abundance of snoRNA known to modify 18S and 28S to be inversely correlated with the abundance of their target. Cyclic patterns in the expression of a number of snoRNA indicate a coordination with rRNA maturation, potentially through an upregulation in their biogenesis, or their release from mature rRNA at the end of the previous cycle of rRNA maturation, in antiphase with the diurnal peak in pre-rRNA. Few cyclic snoRNA have cyclic host genes, indicating the action of regulatory mechanisms in addition to transcriptional activation of the host gene. For highly expressed independently transcribed snoRNA, we find a characteristic RNA polymerase II and H3K4me3 signature that correlates with mean snoRNA expression over the day.

## 1. Introduction

Circadian rhythms in animal physiology and metabolism anticipate predictable diurnal variations in the environment [1,2]. In mammals, the master clock is located in the hypothalamic suprachiasmatic nucleus (SCN) region of the brain. Cells and peripheral organs have autonomous oscillators coordinated with the SCN through hormonal signals [3]. The molecular basis of these cycles is well understood: the Clock:Bmal1 heterodimer activates the transcription of Per and Cry genes, these proteins then repress Clock and Bmal1 transcription [2,3]. Despite Clock:Bmal1 binding its targets in a narrow time window, 6 h after dawn in mouse, the targeted genes peak in expression at varying times [4]. Recent genome-wide sequencing studies of nascent and mature mRNA have shown that rhythmic pre-mRNA transcription is not necessarily followed by rhythmic mRNA levels, and that rhythms in mRNA expression are observed in genes lacking rhythmic transcription [5]. In addition, oscillations in protein levels and phosphorylation states give further evidence for circadian regulation operating at all levels from transcription to translation, splicing and the maintenance of transcript stability [6–10]. Of particular relevance, many proteins involved in ribosome biogenesis have been found to be rhythmic by quantitative proteomics in mouse liver nuclei [10].

Ribosome biogenesis has also been shown to be influenced by the circadian clock through the transcription of translation initiation factors, ribosomal proteins and ribosomal RNAs [11]. For example, in mouse, Rps18 and Rpl30 mRNA expression in the polysomal RNA fraction peaks at 14–22 h after dawn [11]. The 45S pre-rRNA peaks around the middle of the day and is synchronized with ribosomal proteins potentially through UBF1 binding [11]. Ribosome biogenesis consumes a large amount of cellular energy and appears to be diurnally coordinated, possibly to synchronize with nutrient availability [10]. However, little is known about the dynamics of the complex process of rRNA biogenesis across the circadian cycle, or the regulation of the many small nucleolar RNAs (snoRNAs) that support the folding and modification of rRNA precursors.

SnoRNAs are short non-coding RNAs with a conserved role in ribosome biogenesis. SnoRNAs are found in both eukaryotes and archaea indicating an ancient origin [12]. In mammals, many of the currently characterized snoRNA are located in the introns of protein-coding genes, from which they are processed after splicing and debranching of the intron lariat [13]. SnoRNA are also found embedded within annotated non-coding host genes (named processed transcripts in current genome biotype annotations), lincRNAs and in non-genic regions. Two classes of snoRNA have been defined, box C/D and box H/ACA, guiding the methylation of rRNA and its pseudouridylation, respectively. Box C/D snoRNAs have an additional role in the cleavage and folding of pre-rRNA. However, numerous ‘orphan’ snoRNAs outside of these two classes are also known to exist in mammalian genomes, and a variety of novel functional roles for snoRNAs have also emerged. Suggested non-canonical functions of snoRNA include the cross-modification of other snoRNA, binding to other ncRNAs (e.g. 7SK), the editing and splicing of mRNA, association with accessible chromatin and as precursors for miRNA [13]. A potential role for snoRNA in circadian metabolism in mouse and human has also been suggested [14], and snoRNA host genes in *Drosophila* have been shown to oscillate [15] but, to date, the extent of circadian dynamics across the diversity of mammalian snoRNA transcripts is unknown.

Here we take advantage of published nascent (nuclear poly A−) and mature (total poly A+) RNA sequencing datasets [5] to explore the expression dynamics of ribosome biogenesis in mouse liver. We show that nascent RNA-seq data are a rich resource that reveal both snoRNA and pre-rRNA dynamics, and using a new approach to detect periodic expression we reveal novel subpopulations of circadian snoRNA and a distinct subpopulation with time-varying expression greatly in excess of their host genes. Additional data on chromatin state [16] give further novel insights into snoRNA biogenesis. Overall these data suggest that snoRNAs regulated with circadian periodicity are tightly integrated with ribosome biogenesis in mammalian cells.

## 2. Results

We quantify the remarkable variations in snoRNA, host mRNA and rRNA abundance, and explore their interrelationships, in next-generation sequencing data generated across the circadian cycle in mouse liver [5,16]. Cyclical variations in microRNA expression across the circadian cycle have been noted [17,18], as have such variations in lincRNA [5], but to date such changes in snoRNA and rRNA have not been revealed. We adopt the current mouse assembly (Ensembl GRCm38) and annotation (84) for coding and non-coding genes. Approximately 1500 snoRNA genes are included, many from RFAM computational predictions (which have had a controversial status [19]). Thus, we explore the current catalogue of snoRNA gene expression in a diverse range of sequencing data from mouse.

### 2.1. SnoRNA are a major constituent of nascent sequencing data

Nascent sequencing captures nuclear RNA prior to the formation of the 3′-end [5]: samples were DNase-treated and depleted of polyadenylated RNA but rRNA was not removed. The importance of polyA depletion in nascent sequencing is stressed in [20]. Thus, we found the nascent sequencing data contained reads mapping to most RNA species, including those that are not polyadenylated in their mature form such as snoRNA and rRNA. Two biological samples were obtained at six time points from Zeitgeber Time 0 (ZT0, dawn) to ZT20 (20 h after dawn) from 12 different mice (see [5] for details).

**Figure 1.**
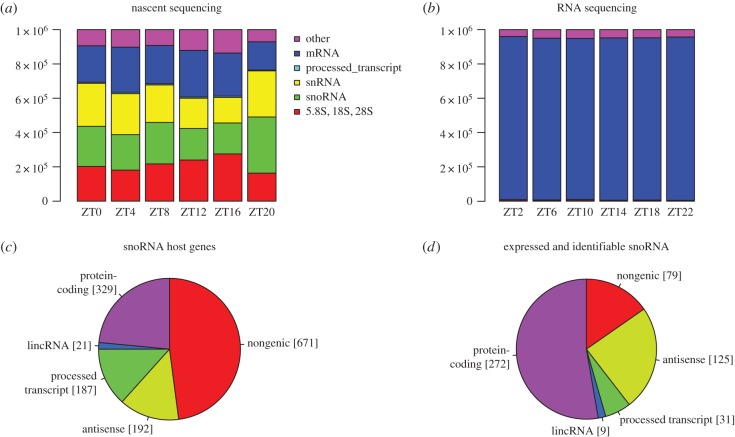
All major categories of non-coding RNA are captured by nascent sequencing. (*a*) Stacked bar charts show the total expression of five selected RNA biotypes in nascent sequencing, and in RNA sequencing data (*b*), at six time points (data from [5]). Quantification is in TPM and hence sums to 10^6^ at each time point. (*c*) Chart depicts the numbers of small nucleolar RNAs annotated in Ensembl classified according to host gene biotype, designated antisense if on the opposite strand to an overlapping gene, else designated non-genic. (*d*) The number of small nucleolar RNAs categorized as in (*c*) that are both expressed and identifiable by a uniquely mapping read in nascent sequencing data.

The abundance of RNA transcripts was quantified in TPM using the Kallisto pseudo-alignment technique [21]. This requires the set of transcripts of interest to be compiled (Ensembl GRCm38) to which we added the 5.8S, 18S and 28S pre-rRNA sequences (snOPY database [22]). The large proportions of snoRNA and rRNA species, and their variation over the day in nascent sequencing data were unexpected but readily apparent (figure 1*a*). It was evident that mRNA constituted between 16% and 27% of the RNA in nascent sequencing across the day, with rRNA, snoRNA and snRNA all accounting for at least 15% of sequenced RNA. By contrast, mRNA constituted over 94% of RNA abundance in conventional RNA sequencing data (figure 1*b*).

### 2.2. SnoRNA hosted by protein-coding genes and non-genic snoRNA are extensively expressed in mouse liver

There is a considerable discrepancy between the number of snoRNA genes curated in the literature and the number annotated as snoRNA in Ensembl, based on a computational prediction protocol. The number of snoRNA genes in mammals has been estimated as 216 (*H. sapiens*) [23], which is only a fraction of the 1484 snoRNA annotated in the mouse assembly GRCm38 (mm10). To address whether these genes are expressed in mouse liver, for each of the 12 sequencing datasets, we selected a lower threshold of TPM expression as the first quartile (0.7–1.6 TPM) and considered all genes with expression above this threshold in any dataset to be expressed. As an additional test, we required at least a single uniquely mapping read per transcript to call a snoRNA identifiable.

The categorization of all Ensembl snoRNAs according to the gene type of the host gene (if any), and the numbers expressed in nascent sequencing are indicated in figure 1*c*,*d*. Overall, we found 516 snoRNA (37%) to be both expressed and identifiable in mouse liver. For snoRNA hosted by protein-coding genes the fraction rises to 83%. A smaller proportion of antisense snoRNA were expressed and identifiable, but the fraction was still surprisingly high at 65%. By contrast, only 12% of other non-genic snoRNA meet these criteria. As this analysis was based on the alignment of reads to sequences, duplicate sequences had to be eliminated (101 snoRNA genes had one or more duplicates and were replaced by 17 exemplars to give a total of 1400 unique genes). Electronic supplementary material, file S1, lists the snoRNA in mm10 along with their locus, that of their host, RFam family, snoRNA type, equivalence class and whether expressed or not.

We then examined whether non-genic snoRNA tend to have higher sequence similarity with other snoRNA as an explanation of their lack of identifiability. Using Blast, we built sets of genes with sequence alignments from 85 to 95%, and identified genes with 100% sequence identity to another gene and found that snoRNA with processed transcript hosts were more prevalent than expected in the 85% similarity set and that non-genic snoRNA were not (electronic supplementary material, figure S1). The number of identifiable genes (those with uniquely mapping reads) reduced as sequence similarity increased, and in the 85% similarity set antisense snoRNA were more identifiable than expected and non-genic snoRNA less so (electronic supplementary material, figure S1). However, at 85% similarity, only 71 non-genic genes were not identifiable, and so sequence similarity appeared to be only a small factor in the eightfold reduction in the number of non-genic snoRNA that were actually expressed and identifiable: This class of snoRNA does not appear to be active in mouse liver.

### 2.3. A subpopulation of snoRNA have time-varying expression greatly in excess of their host gene

**Figure 2.**
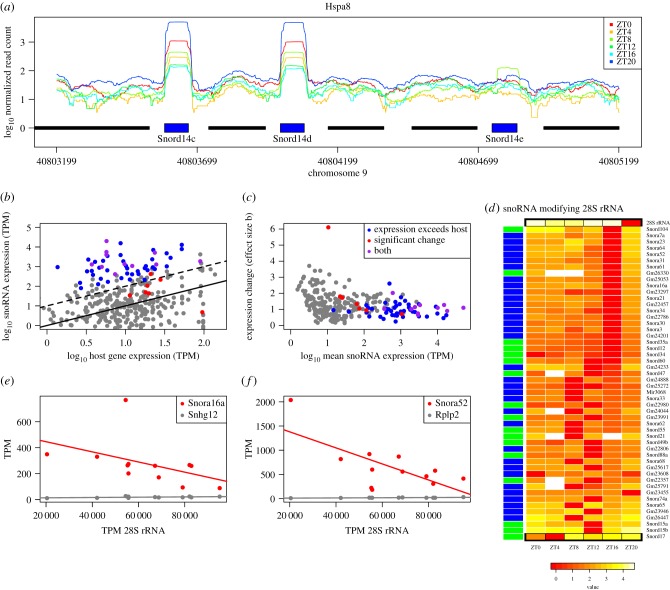
SnoRNA expression varies considerably over time. (*a*) Normalized read depth in nascent sequencing data over the Hspa8 locus at six time points. The locations of Hspa8 exons are shown by black bars, snoRNA by blue bars. Nascent sequencing depth was 70M–157M and coverage was normalized to 10^8^. (*b*) Scatterplot of snoRNA expression against host gene expression at ZT0. Points above the solid black line represent snoRNA with abundance greater than their host, and those above the dashed black line have expression 10 times greater than their host. (*c*) Scatterplot of log fold change in snoRNA expression (b value calculated by sleuth) against mean expression over the time series. In (*b*,*c*), blue symbols indicate snoRNA with expression at least 10 times that of their host gene at all time points, red indicates a significant change in expression (adjusted *p* ≤ 0.05), and purple shows snoRNA satisfying both criteria. (*d*) Heatmap of the expression of 28S rRNA and snoRNA known to modify 28S. Scale is log_10_ difference in TPM from minimum. 28S rRNA (top row) has peak expression at ZT12–ZT16, whereas snoRNA known to interact with 28S have minimum expression at this time. Box H/ACA snoRNA are indicated by blue side colours and box C/D by green. (*e*,*f*) Scatterplots of the expression of selected 28S-modifying snoRNA against 28S expression for the 12 samples available (two replicates at six time points). (*e*) Scatterplot of Snord92 (host gene Wdr43) and (*f*) Snora52 (host gene Rplp2) against 28S expression; lines show linear regressions for snoRNA (red) and host gene (grey).

The difference in expression between genic snoRNA and their host genes was a striking feature of the nascent sequencing data. For example, Snord14c and Snord14d were 20 times more highly expressed than their host Hspa8 at certain times of the day (figure 2*a* and electronic supplementary material, figure S2). Many snoRNA were consistently more highly expressed than their nascent host gene; indeed, 56 were at least 10 times more greatly expressed than their host gene at all time points (figure 2*b*). To assess the change in expression in these genes, we might consider the fold change between maximum and minimum values over the time course. However, simply requiring a threshold of a twofold change in mean expression would lead to the conclusions that 63% of all snoRNA with a host showed differential expression, and that a comparable fraction (66%) of snoRNA with expression in excess of their host were differentially expressed. In fact, testing for differential expression using the Wald test (implemented in sleuth [24]) such that variability between replicates is accounted for led to a very different conclusion: 4% of snoRNA with a host (21 genes) showed significant changes and 25% of snoRNA with expression in excess of their host were differentially expressed. The 21 genes identified had adjusted *p*-values ≤0.05 after accounting for the testing of 98 327 transcripts, and the same set were significant if we considered only snoRNA and chose a conservative threshold of 0.005 after Benjamini–Hochberg correction (a conservative threshold is warranted to account for the selection of minimum and maximum values over the time course). The extent and significance of the fold changes in snoRNA expression over the day are indicated in figure 2*c* by the plot of effect size (the b value computed by sleuth, proportional to log fold change) over the time course against mean expression. Known modifiers of 28S are among the 14 genes satisfying both criteria in figure 2*b*,*c*: Snord17, Snora23, Snora65, Snora74a and Gm23946. (Electronic supplementary material, file S2, lists these genes and provides their expression data.) These properties of snoRNA abundance raise questions as to the relationship between snoRNA and the rRNA they modify, and raise the possibility that some snoRNA may be cyclically expressed. The limitations of assessing circadian regulation through comparisons of maximal and minimal expression are also evident and we address these below.

### 2.4. The expression of snoRNA known to modify 18S and 28S rRNA is negatively correlated with rRNA expression

To obtain a reliable functional annotation of snoRNA, we found exact sequence matches for Ensembl genes in the snOPY database [22] and thereby accessed curated data on the modification of rRNA by snoRNA. This resource also provided informative names for many mouse genes whose names in Ensembl begin ‘Gm’ (following snOPY usage, these names are capitalized). Using this information, we observed many snoRNA known to modify 18S and 28S rRNA to have minimum expression at ZT12 or ZT16, that is, precisely the time when 18S and 28S expression reached a peak (figure 2*d* and electronic supplementary material, figure S3) and to increase thereafter. To quantify this unexpected relationship, we derived linear models for the expression of each snoRNA as a function of 28S expression, and similarly for the host genes of these snoRNA and 28S expression. The scatterplots of figure 2*e*,*f* illustrate two examples where snoRNA expression is negatively correlated with 28S and the host gene is positively correlated with 28S. To assess the statistical significance of these correlations, we compared the number of snoRNA targeting 28S that are negatively correlated with 28S with the numbers negatively correlated in the remainder of expressed genes at a specified value of *R*^2^ using the hypergeometric test (and similarly with positively correlated genes, and for host genes). Rather than select a value of *R*^2^ *a priori*, we assessed overrepresentation for *R*^2^ from 0 to 1, and found the negative correlation of snoRNA to be significant up to an *R*^2^ of 0.56 (*p* = 0.009). The fractions of snoRNA and host genes with positive and negative correlations to 28S are plotted in electronic supplementary material, figure S4, where it can be seen that as *R*^2^ increases the number of genes reaching this level of correlation reduces until there are insufficient genes to test. A similar pattern is found for snoRNA modifying 18S. It should be noted that to counteract the variation in total rRNA, rRNA genes were removed and the expression of other genes rescaled to 10^6^ in the above analysis. This analysis was repeated by quantifying counts of uniquely mapping reads (see Material and methods) and again we saw a striking increase in counts at ZT20 in comparison with ZT16 (electronic supplementary material, figure S5).

A positive correlation between nascent mRNA and pre-rRNA potentially reflects coordinated transcriptional regulation as reported for ribosomal protein genes [11]. By contrast, the negative correlation between snoRNA and pre-rRNA expression implicates post-transcriptional mechanisms that may include intra-nuclear trafficking and release from the ribosome precursor.

### 2.5. Inference of circadian rhythms: a novel method combining residual error and standard deviation of phase

**Figure 3.**
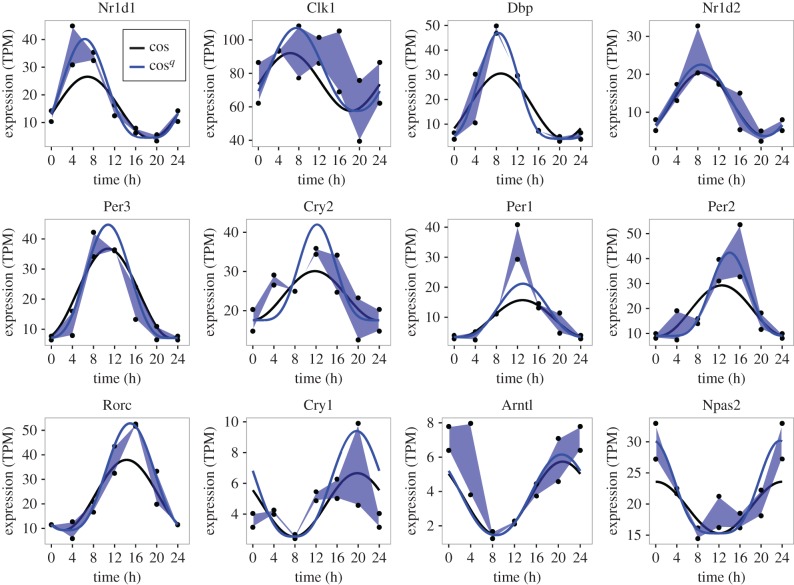
A novel cosine model better fits nascent sequencing data for known clock genes. Expression of 12 established clock genes plotted as a polygons (blue shaded areas) between the maximum replicate data values across the time series and their minimum. Black symbols are data values. The solid lines are the best fit of the standard cosine model (black) and the cosine^*q*^ model (blue) to the median of the replicates at each time point.

To further analyse potential rhythmic oscillations in snoRNA and host genes, we adopted an established false discovery method based on Fourier analysis named F24 [25] (as used in [5]) as an initial filter. Genes with *p*-value for their F24 statistic of greater than 0.2 were not considered further. Exploring alternative mathematical models of circadian dynamics we found that nascent expression data were better fitted by a cosine function raised to a power, creating a more peaked cycle, than a simple cosine function. The improvement in fit of the new model in comparison with the standard cosine model for 12 clock genes is illustrated in figure 3. When assessing the goodness of fit of circadian models to data, we found it important to account for the variability between replicates both in the computational analysis and in visualization. The variability in expression between replicates across the time series is readily perceived by plotting curves between the upper values across the time series, and similarly between the lower values, forming a polygon (as in figure 3). The cosine models were fitted to the median of the replicates at each time point and so the cosine curve would ideally be equally spaced between the upper and lower replicates at each time point. Recall that each data point is from a different mouse, hence the importance of accounting for biological variability.

This modified cosine model with period 24 h, with period 12 h and a linear model were fitted to each time series to assess the fit of a true circadian rhythm, a rapidly oscillating signal (likely noise given the sampling frequency of these data, but potentially due to transcription factor binding [26]) and a gradual change in expression respectively. The Bayesian evidence for each of the three models was calculated using nested sampling [27,28] and time courses were designated circadian where the evidence for the 24 h cycle was 10 times that for the alternative models. The nested sampling algorithm infers the phase and its standard deviation, both of which are of interest in assessing rhythmic behaviour. The likelihood function accounts for the consistency between replicate data, giving less weight to times where replicates differ more (see Material and methods).

In line with comparable methods, 9% of protein coding genes were found to be circadian. To compare the results of our method with published results in more detail, the phase calculated by nested sampling is plotted against the phase calculated by the Fourier method in electronic supplementary material, figure S6*a*, for protein-coding genes designated circadian in [5] (*R*^2^ = 0.53, *p*≤2 × 10^−16^). To further refine the set of circadian genes, those whose phase could not be inferred accurately, or whose fit to the cosine model was less good (as determined by the standard deviation of the phase and the residual (L1) error, respectively, see Material and methods) were excluded. As these two measures can be traded off, we defined a radial score that combines them, and excluded the worst scoring 5% of these circadian genes (electronic supplementary material, figure S6*b*). The distribution of phase values by our method and by the published method (where both the quantification of expression and phase calculation differ) are comparable (electronic supplementary material, figure S6*c*). The range of values chosen for the power parameter (*q*) in the proposed cosine model is shown in electronic supplementary material, figure S6*d*. Values of *q* > 1, the value of the standard model, were chosen extensively. Plots of nascent and RNA sequencing data and the fitted models for 12 clock genes can be found in electronic supplementary material, figure S7. Turning to snoRNA and their host genes, the filtering and selection procedure yielded 43 circadian snoRNA and 26 circadian host genes (electronic supplementary material, figure S6*e*). The absolute radial score threshold determined from circadian genes was also applied in this case.

### 2.6. A subpopulation of snoRNA show cyclical expression

**Figure 4.**
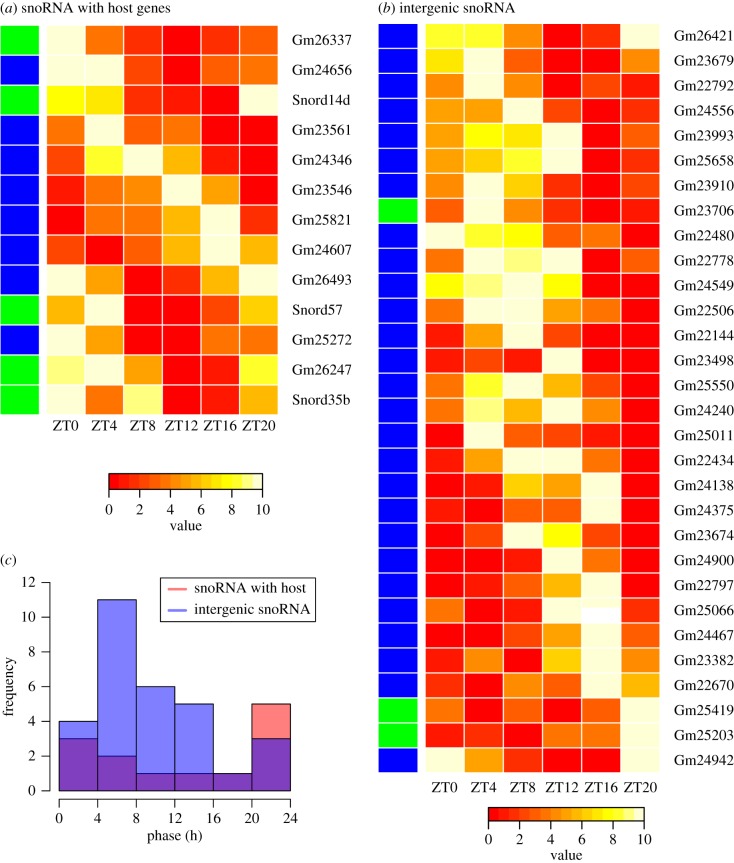
Cyclically expressed snoRNAs. (*a*) Heatmap of the expression of 13 snoRNAs with host genes that are inferred to be cyclic. (*b*) Heatmap of the expression of 30 cyclic intergenic snoRNAs. Heatmap rows are ordered by the inferred phase of the cosine function, box H/ACA snoRNA are indicated by blue side colours, and box C/D by green. Expression is scaled to range from 0 to 10 (see Material and methods). (*c*) Histogram of the phase of snoRNA in (*a*) and in (*b*).

Thirteen snoRNA located in introns were found to be cyclically expressed, including Snord35b, Snord57 and Snord14d. The peak expression of these snoRNA occurred across the day with some preference for the beginning or end of the day (figure 4*a*). Thirty non-genic snoRNA were cyclically expressed, showing peak expression within a more defined period 4–16 h after dawn (figure 4*b*). The distribution of phase values (figure 4*c*) illustrates the differing peak times of these two populations of snoRNA. We next looked for cyclically expressed host genes in both nascent sequencing and RNA sequencing data and identified 26 and 14 cyclic host genes, respectively (electronic supplementary material, figure S8). Of the 30 snoRNA whose host showed cyclic expression in nascent sequencing data, two were found to be cyclically expressed and we observed one of these to be in anti-phase with its host and the other to be in phase (electronic supplementary material, figures S9 and S10). Thus, we found only minimal overlap between snoRNA and host expression patterns possibly indicating that their cyclic behaviour is regulated by mechanisms in addition to transcriptional activation. The model parameters for cyclical snoRNA and their host genes can be found in the electronic supplementary material, file S3.

As is apparent from figure 4, few of the cyclical snoRNA are currently designated ‘Snora’ or ‘Snord’ which indicates a lack of recognition of their status in mouse. However, from the snOPY database we identified SNORA21 (Gm25821), SNORA46 (Gm26493), SNORD88 (Gm26247), SNORD115 (Gm26337) and three SNORA17 genes (Gm25272, Gm24607 and Gm24656) among the cyclic snoRNA with host genes. Considering cyclic snoRNA without host genes, we identified SNORA63 (Gm23679), SNORA71 (Gm22797), SNORD86 (Gm23706) and seven SNORA17 genes (Gm22778, Gm26421, Gm23910, Gm24375, Gm24556, Gm23674 and Gm22670). Of note, genes in the SNORD88 and SNORD115 families are associated with the regulation of splicing [13].

The abundance of cyclic snoRNA was on average 1.5 times that of their host genes. Of these genes, only SNORA46 (Gm26493) was among the set of snoRNA with consistently high ratios of expression relative to their host (at least 10 times greater). None of the cyclic snoRNA were among those found to have statistically significant fold changes (figure 2*b*), thus these populations of snoRNA were disjoint.

### 2.7. 18S and 28S rRNA are cyclically expressed

**Figure 5.**
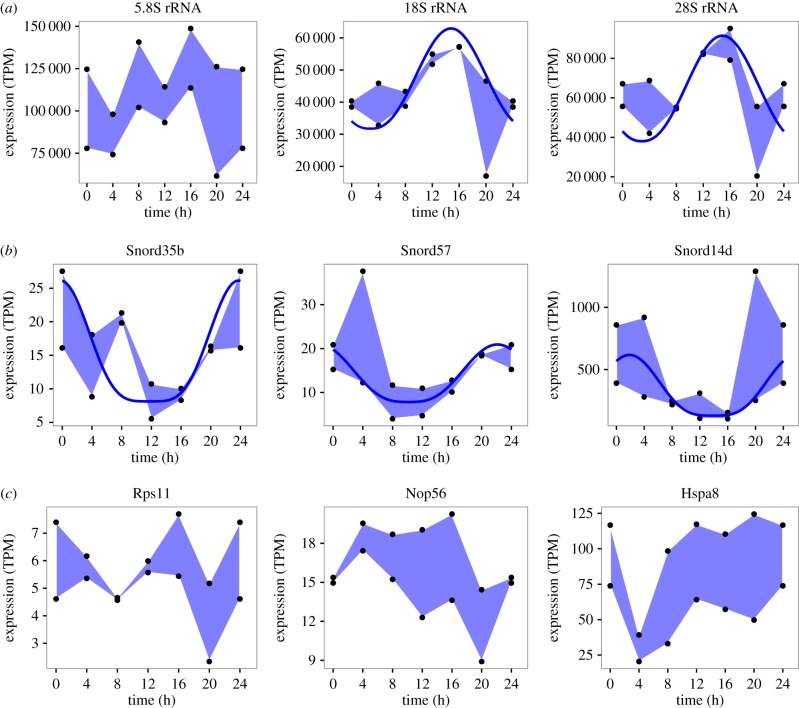
Ribosomal RNA, snoRNA and host gene expression. Expression of 5.8S, 18S and 28S rRNA (*a*), selected cyclic snoRNAs (*b*) and their respective host genes (*c*; Rps11 hosts Snord35b, Nop56 hosts Snord57 and Hspa8 hosts Snord14d) plotted as polygons (blue shaded areas) between the maximum replicate data values across the time series and their minimum. Where expression was inferred to be cyclic, the best fitting cosine^*q*^ model is indicated by a solid blue line. Notably, Snord57 modifies 18S and the protein of its host Nop56 is a component of the box C/D ribonucleoprotein complex.

Applying the circadian modelling introduced above, we next determined that the temporal variations noted earlier in both 18S and 28S rRNA were indeed circadian, while 5.8S expression dynamics did not pass the initial false discovery filtering step. The cyclical patterns of these transcripts are shown in figure 5*a* along with selected circadian snoRNAs (Snord35b, Snord57 and Snord14d; figure 5*b*) and their respective host genes (figure 5*c*). Snord57 and Snord14d are known to modify 18S rRNA and it is readily seen that their expression profiles show starkly contrasting phase.

Of the 10 cyclic snoRNA with host genes that had matches in the snOPY database, four modify 28S: Snord35b, SNORA21, SNORA17 (Gm25272) and SNORD88. Three modifiers of 18S were found among the cyclic snoRNA with hosts: Snord57, Snord14d and SNORA46. Thus, we found the majority of cyclic snoRNA with host genes to be associated with rRNA modification; however, as the majority of these genes modify 28S or 18S this number did not constitute a statistical enrichment. The cyclic snoRNA without hosts we found included SNORA17 (Gm24375) a modifier of 28S, and SNORA71, a modifier of 18S.

### 2.8. Highly expressed non-genic snoRNA have a distinct chromatin signature

Histone modifications H3K4me3 and H3K27ac have been shown to vary rhythmically around gene promoters in mouse liver [17], and the rhythmic recruitment of PolII at the promoter has been demonstrated to oscillate in phase with RNA polymerase II (PolII) levels on the gene body indicating that it is the recruitment of PolII rather than its release that is critical to diurnal transcription [16]. A set of strong circadian promoters has been proposed to drive circadian genes with high amplitude and high average expression, and is associated with high paused PolII levels (relative to H3K4me3) and the extension of H3K4me3 into the gene body [29]. While we are interested to discover whether the cyclic non-genic snoRNA discovered above have a cyclic chromatin environment that would explain these variations, we do not limit our analysis to these genes but consider all non-genic snoRNA.

**Figure 6.**
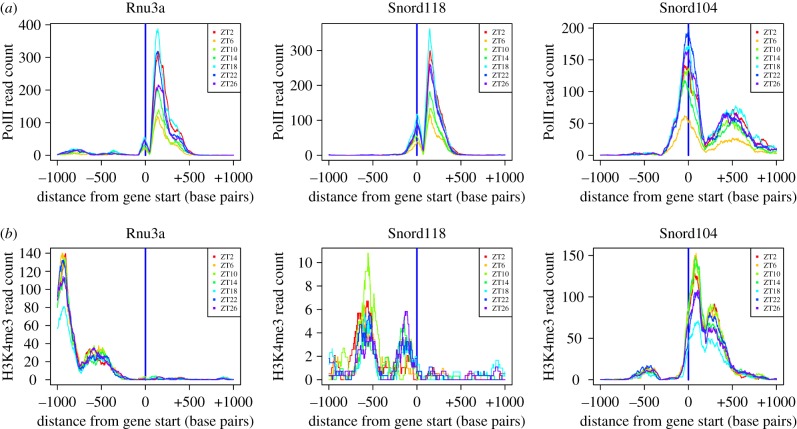
Independently transcribed snoRNA have a distinct chromatin signature. Normalized read depth in a 2 kb region centred on the snoRNA gene start, and oriented in the direction of transcription, is shown for selected independently transcribed snoRNA for RNA polymerase II (*a*) and for H3K4me3 ChIP sequencing data (*b*) at seven time points (data from [16]). A peak in PolII over the gene and an adjacent peak in H3K4me3 are characteristic chromatin features.

To investigate whether non-genic snoRNA have a chromatin signature that might support their transcription as independent genes, and to explore any temporal variations indicative of circadian expression, we mapped the PolII and H3K4me3 time-series data from mouse liver published by Le Martelot [16] and located peaks at each time point, and in the combined data using MACS2 [30]. Beginning with clock genes, the abundance of PolII and H3K4me3 around clock gene promoters, and the variation in these signals is shown in electronic supplementary material, figure S11, for Per2 and Nr1d1. Consistent with previous studies, a substantial peak in PolII was observed at the gene start with peaks in H3K4me3 downstream. Of the 12 clock genes examined, PolII levels decreased towards background levels at one or more time points in three cases (Per2, Dbp and Npas2), whereas H3K4me3 levels remained above background across the day in all cases. We then examined the chromatin signature of three snoRNA known to be independently transcribed, namely, Rnu3a (U3), Snord13 and Snord118 [23,31], and found a distinctive peak in PolII at the gene start in all three cases (figure 6 and electronic supplementary material, figure S12). A considerable temporal variation in this signal was also apparent. These genes overlapped with peaks in PolII and H3K4me3 called by MACS2 and so we searched for other non-genic snoRNA that shared these properties and found six: Snord104 and SNORA76 (Gm22711) (which are clustered as in human [23]), Snora57 (reported to be monocistronic in [22]), Snora17, Gm25501 and Gm23596 (which are antisense to Ank2 and intergenic, respectively). In the cases of Rnu3a and Snord13, the upstream peaks in H3K4me3 were over the start of an adjacent gene on the opposite strand (Gtf3c6 and Tti2, respectively). Although Snora17 has no annotated host gene in the release of Ensembl we have adopted, it overlaps Snhg7 in Refseq. The major peaks in the chromatin signals around Snora17 were located over the Refseq host gene start (with minor peaks over the gene itself) which support the existence of the host.

The eight snoRNA we characterize as independently transcribed had higher PolII, H3K4me3 and nascent sequencing expression than did non-genic snoRNA that lack overlapping MACS2 peaks in PolII and H3K4me3 (*p* ≤ 2.7 × 10^−4^ by Wilcoxon test). The input PolII and H3K4me3 levels of these genes did not differ from that of the remaining non-genic snoRNA (to determine an overlap the snoRNA gene locus was extended by 200 bases, and the expression of these extended features was quantified in RPKM). It is readily evident in electronic supplementary material, figure S13, that these eight genes form a distinct cluster of highly expressed snoRNA with corresponding chromatin marks. In addition, we found Snord60 and Snora78 (which overlap short antisense transcripts Rab26 and Snhg9, respectively) to have similar chromatin signatures.

The PolII signal of each of the eight independently transcribed snoRNA had a distinct minimum at ZT6, and for all except Rnu3a there was a dip in nascent sequencing expression at ZT8 relative to ZT4, followed by an increase at ZT12 (electronic supplementary material, figure S14) and variable expression thereafter. The differing sampling times of these data made the assessment of any correlations unreliable. The minimum PolII signal was at least twice the background, and the log2 fold change of the maximum signal (relative to the same background) was at least 1.4 greater than the minimum which again indicated a notable temporal variation. Snord13 was the most circadian with a F24 FDR 0.12 (three other genes also had *p* < 0.2). Our Bayesian method could not be applied to the chromatin data as there were no replicates. None of these snoRNA had cyclic expression in the nascent sequencing data. However, the log2 fold change in nascent expression was in the range 0.9–2.9 when maximum and minimum expression over the day were compared, and therefore temporal variation was evident in all cases. The H3K4me3 signal dipped at ZT14 or ZT18 in six cases but with less pronounced fold changes over background than for PolII (electronic supplementary material, figure S14). Applying the F24 FDR test, we found three snoRNA to have *p*-values for H3K4me3 expression less than 0.2 (Rnu3a, Snord104 and Gm22711).

## 3. Discussion

Using novel computational statistical techniques, we have uncovered previously unrecognized patterns in the abundance of nuclear pre-rRNAs and snoRNAs, and correlations between them. A population of snoRNA that were at least 10 times as abundant as their nascent host gene, some with statistically significant diurnally varying expression (but not fitting the cosine function taken as the model for a circadian rhythm) was identified. The expression of snoRNA that modify 18S and 28S was typically in antiphase with that of the target rRNA precursor, as evidenced by negative correlations in abundance.

We found the expression of ribosome precursors 18S and 28S rRNA to follow a circadian rhythm in mammalian liver, peaking at ZT16 and that snoRNA including Snord14d, Snord35b and Snord57 also had cyclical expression patterns in this tissue. Snord57 is known to modify 18S and the protein of its host gene, Nop56, is a component of the box C/D ribonucleoprotein complex. Indeed, proteins Nop56 and Fbl of this complex were found to be cyclical in recently published data [10], with minimum expression at ZT15 and ZT18, respectively, indicating a temporal variation that is comparable with that of many snoRNA that modify 18S and 28S rRNA (figure 2). Thus, there may be common underlying regulation that we are now beginning to unravel. The scope for confirmation of our findings in other time course data was limited as gene expression is typically measured by microarray, or by poly A+ and rRNA depleted RNA sequencing. However, a small number of microarray probes in [11] did match snoRNA and the expression of three cyclical snoRNA was reproduced (electronic supplementary material, figure S15).

The intersection between circadian snoRNAs and circadian host genes was minimal as only two cases were found. In the first, snoRNA and host expression were in antiphase, in the second, expression was in phase. Given the overall proportions of cyclic genes in these categories, there was no enrichment for cyclic host genes among cyclic snoRNAs, hence no evidence for cyclic transcription as the key regulator. As for messenger RNA [2,5], mechanisms in addition to transcription must contribute the regulation of cyclic nuclear snoRNAs.

The correlation of snoRNA host gene and rRNA expression may be the result of co-regulation with rRNA as reported for ribosomal protein genes [11]. An antiphase relationship between many snoRNA and their pre-rRNA target is more surprising, and may show an upregulation of snoRNA biogenesis in anticipation of the increased rRNA levels that peak around ZT16, or may be due to a release (or relocation) of snoRNA from the previous cycle of rRNA maturation that restores their abundance in the nucleus. The peaks in 18S and 28S rRNA after dusk (lights out) are consistent with previous findings of diurnal 45S rRNA synthesis, and coordinated ribosomal protein dynamics in the nucleus that occur during the active period of the day when feeding takes place [10,11]. Such a coordination would provide the energy for ribosome assembly.

Mature snoRNA are concentrated in the nucleolus; however, they undergo extensive intranuclear trafficking during biogenesis [32]. Indeed, the box C/D motif functions as the nucleolar localization signal [33]. In addition, snoRNAs have been found to be involved in splicing outside of the nucleolus [34]. Human U8 (SNORD118) snoRNA precursors have been found in cytoplasmic extracts in levels comparable with those in nuclear extracts [35], but this does not appear to be a typical biogenesis pathway [36]. Thus, for a number of snoRNA, variation in abundance may be attributed in part to cytoplasmic trafficking, and possibly to trafficking between nuclear structures, as well as to their established role in rRNA biogenesis.

Little is known about the role of the chromatin environment as a potential regulator of independently transcribed snoRNA. We found peaks in RNA polymerase II over the gene locus and adjacent peaks in H3K4me3 to be signatures of independently transcribed snoRNA, and, in addition, mean PolII and H3K4me3 levels correlated with mean snoRNA transcript abundance. Time-varying but noncyclic patterns were found in these chromatin marks, with a distinct dip in PolII at ZT6 that may indicate a common regulatory input for this class of snoRNA.

Differences in phase of clock-regulated genes in different organs have been reported [3,14,37], offering insights into the coordination of the peripheral clocks. Our methodology is particularly suited to such investigations as it yields standard deviations for key model parameters such as phase, and the potential to model multiple datasets in an integrative manner.

## 4. Material and methods

### 4.1. Definition of cosine models

Circadian rhythms were modelled by a cosine function that varied between 0 and the maximum expression *a*, with peak expression (i.e. phase) *p* minutes after time 0, raised to the power *q* as follows:4.1
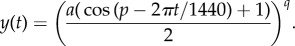


Parameters *a*, *p* and *q* were constrained by the following prior ranges:
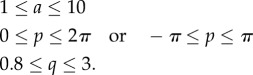


All time-series data were scaled such that the minimum median value was 0 and the maximum median was 10, hence *a* could be at most 10. Two alternative constraints on *p* were used to ensure that the fitted value of this parameter did not lie at the end of the prior range. This might occur for *p* close to 0 or 2*π* in which case the alternative prior centred on 0 (2*π*) was used −*π* ≤ *p* ≤ *π*.

The fit between the cosine models and expression data was assessed using the nested sampling algorithm to calculate the log of Bayesian evidence (also known as the marginal likelihood), log *Z* [27] from the likelihood function and the prior. All priors were selected uniformly from a range bounded by maximum and minimum values given above. A likelihood based on the l1-norm was defined by equations (4.2) and (4.3) [38]. Equation (4.2) defines the normalizing constant *ε*_*t*_ as the expected value of the moduli of the difference between the replicate observations at time *t* (*x*_*t*_) and the value predicted by the kinetic model (*μ*_*t*_). The product of the probabilities of the median observation at time *t* (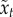) defines the likelihood function for a time series *x* of *m* samples (equation (4.3)). Maximization of this likelihood minimizes the sum of the moduli of the residuals (rather than their squares) on the basis that the testable information is restricted to the expected value of the modulus of the difference between theory and experiment. Should we know both the mean and variance, maximum entropy considerations would lead instead to the Gaussian distribution [38].4.2
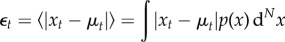
and4.3
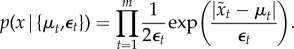


Bayesian evidence values and model parameter estimates (and their standard deviations) were computed using nested sampling for each time series that passed an initial FDR test (the F24 test [25] with *p* ≤ 0.2). A cosine model with a 12 h period and a linear model were also fitted to each time series. Time series where the log *Z* for the 24 h cosine model was 10 times greater than that for the alternative models were considered circadian if, in addition, they passed a test on the standard deviation of phase and L1 error. The threshold for the final radial score test was derived empirically from genes found to be circadian in earlier studies [5]. R code for nested sampling is provided in electronic supplementary material, file S4.

### 4.2. Processing of sequencing data

The gene annotation file for GRCm38 was downloaded from Ensembl (version 84) and processed with bedtools [39] and in R to identify snoRNA, their locus, snoRNA host genes and their locus, and gene biotypes. SnoRNA–host gene assignments were reviewed manually using the IGV genome browser. Additional data on RFam families were downloaded from the EBI, and data from the snoPY database [22] were also used. A blast database was created from a fasta file of all snoRNA sequences (using parameters -in snoRNA.fasta -input_type fasta -dbtype nucl -title snoRNAdb -out snoRNAblastdb) and this file was queried using blastn (with parameters -query snoRNA.fasta -db snoRNAblastdb -outfmt 6). The blastn output was further processed in R to obtain data on sequences at 85%, 90% and 95% similarity in addition to those with sequence identity (electronic supplementary material, figure S1).

Nascent and RNA sequencing time-series data were downloaded from GEO GSE36916 [5]. Coding and non-coding transcripts for mouse genome GRCm38 were downloaded from Ensembl to which the 5.8S, 18S and 28S pre-rRNA sequences were added from [22] to create an index file for quantification in TPM using Kallisto [21]. The database consisted of 98 327 transcripts (38 080 genes), and included all protein-coding transcripts, snRNA, lincRNA, scaRNA, processed transcripts, snoRNA plus the three pre-rRNA. As the data in [5] comprised single reads, the effective length parameter was set manually. The length distributions of snoRNA and snoRNA host genes were very different, median lengths 127 and 947 bases, respectively. Hence we set the effective length parameter to minimize the possible inflation of TPM for shorter transcripts (using parameters -single -l 40 -s 200). The Kallisto index was built with kmers of length 19. TPM values for genes were summed from those of their transcripts. Reads were also mapped to Ensembl GRCm38 using bowtie2 (using parameters -L 18 -N 1 -k 20; electronic supplementary material, figure S2) [40]. Uniquely mapping reads were extracted using samtools [41], and unique read counts for snoRNA genes found using htseq-count [42]. These counts were used to determine snoRNA identifiability. Read pileups (figure 2) were created from multiply mapped reads using bedtools with output files subsequently processed in R.

Following [5], the F24 test [25] was applied to the nascent and RNA time-series data by concatenating first and second replicates to create a series from ZT0 to ZT44. We constructed the replicated time series in the same manner in order to have a sample at ZT24 while not duplicating the ZT24 sample alone (electronic supplementary material, figures S3 and S5, show ZT0–ZT24 only).

PolII, H3K4me3 and input time-series data were downloaded from GEO GSE35790 [16]. Reads were mapped to Ensembl GRCm38 using bowtie2 (using parameters -k 2) and uniquely mapping reads were extracted using samtools. MACS2 [30] was used to find peaks in uniquely mapping PolII and H3K4me3 reads at each time point, and in the combined data. Peaks found in the combined data appeared most robust and were intersected with snoRNA locus using bedtools. Read counts and pileups for genomic features were obtained using bedtools and output files were subsequently processed in R (electronic supplementary material, figures S6, S9–S11).

## Supplementary Material

SnoRNA locus and expression.

## Supplementary Material

SnoRNA and host gene expression.

## Supplementary Material

Circadian snoRNA.

## Supplementary Material

R code for nested sampling.

## Supplementary Material

Supplementary Figures

## References

[1] Staiger D, Shin J, Johansson M, Davis SJ 2013 The circadian clock goes genomic. Genome Biol. 14, 208 (10.1186/gb-2013-14-6-208) 23796230 PMC3706883

[2] Benegiamo G, Brown SA, Panda S 2016 RNA dynamics in the control of circadian rhythm. In RNA processing (ed. Yeo GW), pp. 107–122. Berlin, Germany: Springer. 10.1007/978-3-319-29073-7_5 27256384

[3] Yan J, Wang H, Liu Y, Shao C 2008 Analysis of gene regulatory networks in the mammalian circadian rhythm. PLoS Comput. Biol. 4, e1000193 (10.1371/journal.pcbi.1000193) 18846204 PMC2543109

[4] Rey G, Cesbron F, Rougemont J, Reinke H, Brunner M, Naef F 2011 Genome-wide and phase-specific DNA-binding rhythms of BMAL1 control circadian output functions in mouse liver. PLoS Biol. 9, e1000595 (10.1371/journal.pbio.1000595) 21364973 PMC3043000

[5] Menet JS, Rodriguez J, Abruzzi KC, Rosbash M 2012 Nascent-Seq reveals novel features of mouse circadian transcriptional regulation. eLife 1, e00011 (10.7554/eLife.00011) 23150795 PMC3492862

[6] McGlincy NJ, Valomon A, Chesham JE, Maywood ES, Hastings MH, Ule J 2012 Regulation of alternative splicing by the circadian clock and food related cues. Genome Biol. 13, R54 (10.1186/gb-2012-13-6-r54) 22721557 PMC3446320

[7] Reddy AB *et al.* 2006 Circadian orchestration of the hepatic proteome. Curr. Biol. 16, 1107–1115. (10.1016/j.cub.2006.04.026) 16753565

[8] Mauvoisin D, Wang J, Jouffe C, Martin E, Atger F, Waridel P, Quadroni M, Gachon F, Naef F 2014 Circadian clock-dependent and -independent rhythmic proteomes implement distinct diurnal functions in mouse liver. Proc. Natl Acad. Sci. USA 111, 167–172. (10.1073/pnas.1314066111) 24344304 PMC3890886

[9] Robles MS, Cox J, Mann M 2014 In-vivo quantitative proteomics reveals a key contribution of post-transcriptional mechanisms to the circadian regulation of liver metabolism. PLoS Genet. 10, e1004047 (10.1371/journal.pgen.1004047) 24391516 PMC3879213

[10] Wang J *et al.* 2017 Nuclear proteomics uncovers diurnal regulatory landscapes in mouse liver. Cell Metab. 25, 102–117. (10.1016/j.cmet.2016.10.003) 27818260 PMC5241201

[11] Jouffe C, Cretenet G, Symul L, Martin E, Atger F, Naef F, Gachon F 2012 The circadian clock coordinates ribosome biogenesis. PLoS Biol. 11, e1001455 (10.1371/journal.pbio.1001455) PMC3536797 23300384

[12] Bachellerie J-P, Cavaillé J, Hüttenhofer A 2002 The expanding snoRNA world. Biochimie 84, 775–790. (10.1016/S0300-9084(02)01402-5) 12457565

[13] Dupuis-Sandoval F, Poirier M, Scott MS 2015 The emerging landscape of small nucleolar RNAs in cell biology. Wiley Interdisc. Rev. RNA 6, 381–397. (10.1002/wrna.1284) PMC4696412 25879954

[14] Zhang R, Lahens NF, Ballance HI, Hughes ME, Hogenesch JB 2014 A circadian gene expression atlas in mammals: implications for biology and medicine. Proc. Natl Acad. Sci. USA 111, 16 219–16 224. (10.1073/pnas.1408886111) PMC4234565 25349387

[15] Hughes ME, Grant GR, Paquin C, Qian J, Nitabach MN 2012 Deep sequencing the circadian and diurnal transcriptome of drosophila brain. Genome Res. 22, 1266–1281. (10.1101/gr.128876.111) 22472103 PMC3396368

[16] Le Martelot G *et al.* 2012 Genome-wide RNA polymerase II profiles and RNA accumulation reveal kinetics of transcription and associated epigenetic changes during diurnal cycles. PLoS Biol. 10, e1001442 (10.1371/journal.pbio.1001442) 23209382 PMC3507959

[17] Vollmers C, Schmitz RJ, Nathanson J, Yeo G, Ecker JR, Panda S 2012 Circadian oscillations of protein-coding and regulatory RNAs in a highly dynamic mammalian liver epigenome. Cell Metab. 16, 833–845. (10.1016/j.cmet.2012.11.004) 23217262 PMC3541940

[18] Wang H, Fan Z, Zhao M, Li J, Lu M, Liu W, Ying H, Liu M, Yan J 2016 Oscillating primary transcripts harbor miRNAs with circadian functions. Sci. Rep. 6, 21598 (10.1038/srep21598) 26898952 PMC4761921

[19] Makarova JA, Kramerov DA 2011 SNOntology: myriads of novel snornas or just a mirage? BMC Genom. 12, 543–543 (10.1186/1471-2164-12-543) PMC3349704 22047601

[20] Herzel L, Neugebauer KM 2015 Quantification of co-transcriptional splicing from RNA-Seq data. Methods 85, 36–43. (10.1016/j.ymeth.2015.04.024) 25929182

[21] Bray NL, Pimentel H, Melsted P, Pachter L 2016 Near-optimal probabilistic RNA-seq quantification. Nat. Biotechnol. 34, 525–527. (10.1038/nbt.3519) 27043002

[22] Yoshihama M, Nakao A, Kenmochi N 2013 snOPY: a small nucleolar RNA orthological gene database. BMC Res. Notes 6, 426–426 (10.1186/1756-0500-6-426) PMC4015994 24148649

[23] Dieci G, Preti M, Montanini B 2009 Eukaryotic snoRNAs: a paradigm for gene expression flexibility. Genomics 94, 83–88. (10.1016/j.ygeno.2009.05.002) 19446021

[24] Pimentel HJ, Bray N, Puente S, Melsted P, Pachter L 2016 Differential analysis of RNA-Seq incorporating quantification uncertainty. (http://biorxiv.org/content/early/2016/06/10/058164)

[25] Wijnen H, Naef F, Young MW 2005 Molecular and statistical tools for circadian transcript profiling. Methods Enzymol. 393, 341–365. (10.1016/S0076-6879(05)93015-2) 15817298

[26] Westermark P, Herzel H 2013 Mechanism for 12 hr rhythm generation by the circadian clock. Cell Rep. 3, 1228–1238. (10.1016/j.celrep.2013.03.013) 23583178

[27] Aitken S, Akman O 2013 Nested sampling for parameter inference in systems biology: application to an exemplar circadian model. BMC Syst. Biol. 7, 72 (10.1186/1752-0509-7-72) 23899119 PMC3735395

[28] Aitken S *et al.* 2015 Transcriptional dynamics reveal critical roles for non-coding RNAs in the immediate-early response. PLoS Comput. Biol. 11, e1004217 (10.1371/journal.pcbi.1004217) 25885578 PMC4401570

[29] Westermark P 2016 Linking core promoter classes to circadian transcription. PLoS Genet. 12, e1006231 (10.1371/journal.pgen.1006231) 27504829 PMC4978467

[30] Zhang Y *et al.* 2008 Model-based analysis of ChIP-Seq (MACS). Genome Biol. 9, R137 (10.1186/gb-2008-9-9-r137) 18798982 PMC2592715

[31] Makarova JA, Kramerov DA 2009 Analysis of C/D box snoRNA genes in vertebrates: the number of copies decreases in placental mammals. Genomics 94, 11–19. (10.1016/j.ygeno.2009.02.003) 19272437

[32] Kiss T, Fayet E, Jady B, Richard P, Weber M 2006 Biogenesis and intranuclear trafficking of human Box C/D and H/ACA RNPs. Cold Spring Harbor Symp. Quant. Biol. 71, 407–417. (10.1101/sqb.2006.71.025) 17381323

[33] Samarsky DA, Fournier MJ, Singer RH, Bertrand E 1998 The snoRNA box C/D motif directs nucleolar targeting and also couples snoRNA synthesis and localization. EMBO J. 17, 3747–3757. (10.1093/emboj/17.13.3747) 9649444 PMC1170710

[34] Falaleeva M *et al.* 2016 Dual function of C/D box small nucleolar RNAs in rRNA modification and alternative pre-mRNA splicing. Proc. Natl Acad. Sci. USA 113, E1625–E1634. (10.1073/pnas.1519292113) 26957605 PMC4812717

[35] Watkins NJ, Lemm I, Lührmann R 2007 Involvement of nuclear import and export factors in U8 box C/D snoRNP biogenesis. Mol. Cell. Biol. 27, 7018–7027. (10.1128/MCB.00516-07) 17709390 PMC2168896

[36] Pradet-Balade B, Girard C, Boulon S, Paul C, Azzag K, Bordonné R, Bertrand E, Verheggen C 2011 CRM1 controls the composition of nucleoplasmic pre-snorna complexes to licence them for nucleolar transport. EMBO J. 30, 2205–2218. (10.1038/emboj.2011.128) 21522132 PMC3117649

[37] Hughey JJ, Butte AJ 2016 Differential phasing between circadian clocks in the brain and peripheral organs in humans. J. Biol. Rhythms 31, 588–597. (10.1177/0748730416668049) 27702781 PMC5105327

[38] Sivia D, Skilling J 2006 Data analysis: a Bayesian tutorial. Oxford, UK: Oxford University Press.

[39] Quinlan AR, Hall IM 2010 BEDTools: a flexible suite of utilities for comparing genomic features. Bioinformatics 26, 841–842. (10.1093/bioinformatics/btq033) 20110278 PMC2832824

[40] Langmead B, Salzberg SL 2012 Fast gapped-read alignment with Bowtie 2. Nat. Methods 9, 357–359. (10.1038/nmeth.1923) 22388286 PMC3322381

[41] Li H *et al.* 2009 The sequence alignment/map format and SAMtools. Bioinformatics 25, 2078–2079. (10.1093/bioinformatics/btp352) 19505943 PMC2723002

[42] Anders S, Pyl PT, Huber W 2015 HTSeq—a Python framework to work with high-throughput sequencing data. Bioinformatics 31, 166–169. (10.1093/bioinformatics/btu638) 25260700 PMC4287950

