# Supplementary figures and images for: The circadian dynamics of small nucleolar RNA in the mouse liver
Source: J R Soc Interface. 2017 May 3;14(130):20170034. doi: 10.1098/rsif.2017.0034 (PMC5454292; doi:10.1098/rsif.2017.0034)

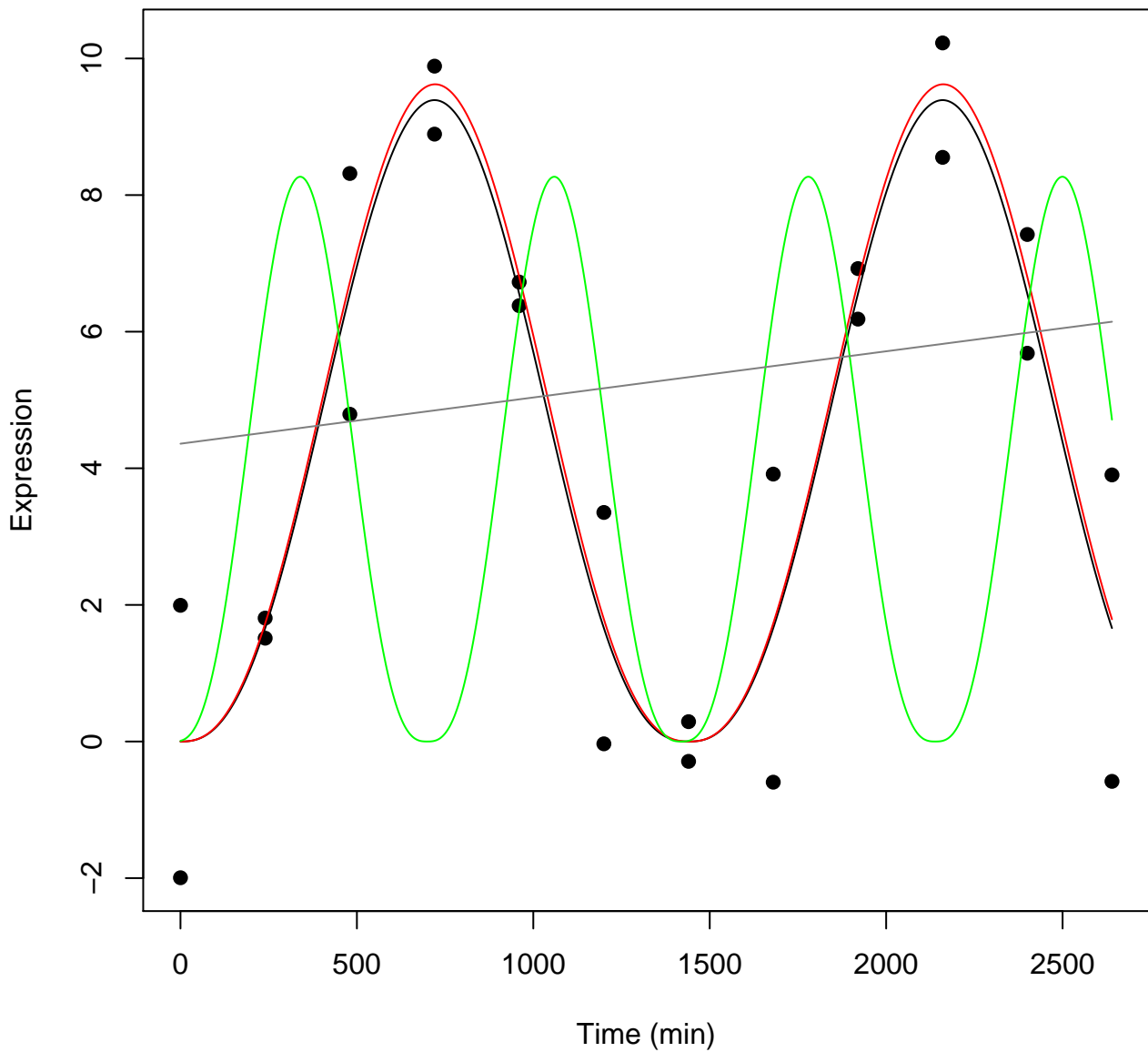

Supplement: R code for nested sampling. [file rsif20170034supp4.gz › R_code/Rplots.pdf]
