# Supplementary material for: The circadian dynamics of small nucleolar RNA in the mouse liver
Source: J R Soc Interface. 2017 May 3;14(130):20170034. doi: 10.1098/rsif.2017.0034 (PMC5454292; doi:10.1098/rsif.2017.0034)
Supplement: Supplementary Figures [file rsif20170034supp5.pdf]

The circadian dynamics of small nucleolar RNA

Stuart Aitken and Colin A. Semple
MRC Human Genetics Unit, MRC Institute of Genetics and Molecular Medicine,
University of Edinburgh, Edinburgh, EH4 2XU, United Kingdom.
E-mail: stuart.aitken@igmm.ed.ac.uk

Supporting Information

Supplementary File 1. SnoRNA locus and expression. A comma separated file listing all snoRNA Ensembl ids in GRCm38, locus, id and locus of host (if any), RFAM family, and whether expressed or not.

Supplementary File 2. SnoRNA and host gene expression. A comma separated file listing snoRNA Ensembl ids for genes identified in Fig 2c and d, log10 ratio to host expression, mean expression, fold change (b value), p and q values from sleuth.

Supplementary File 3. Circadian snoRNA. A comma separated file listing Ensembl ids for circadian genes, circadian model parameters, and F24 values.

Supplementary File 4. R code for nested sampling. A tar archive of the R code for nested sampling with examples of usage.

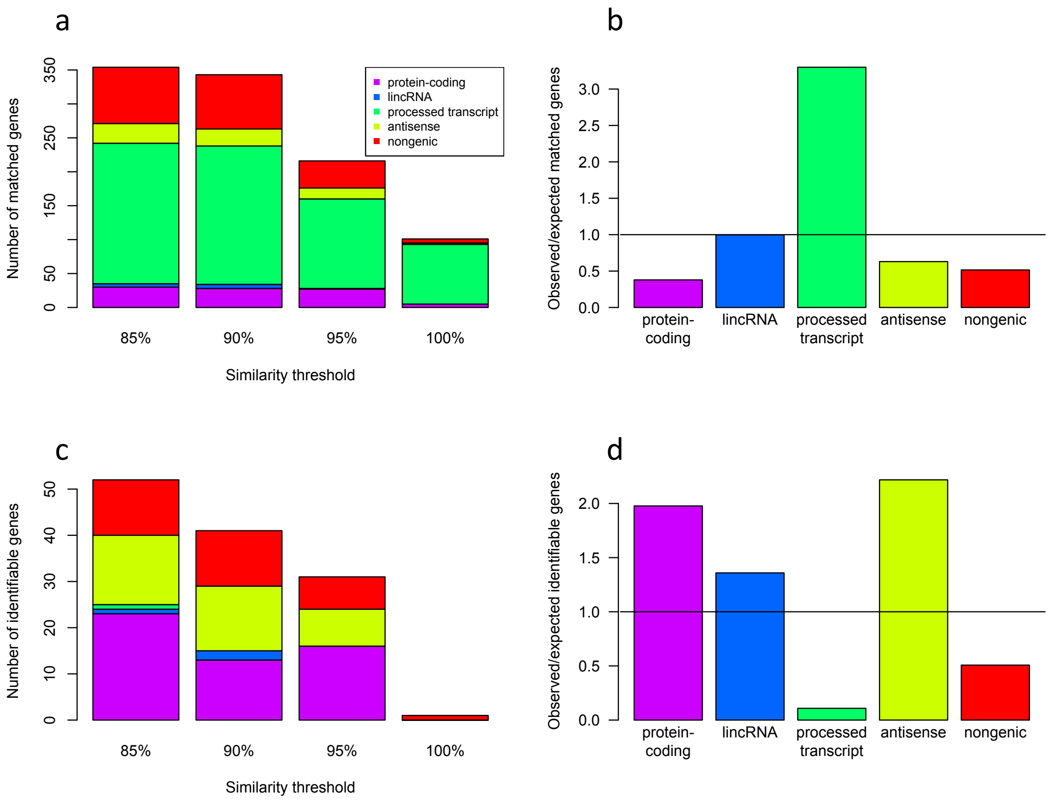

Figure S1: **Similarity and identifiability of snoRNA genes.**
a Stacked bar chart of the numbers of snoRNA grouped into sets of equivalent genes at similarity thresholds from 85% to 100%. These sets of matched genes were built using Blast and required alignments of at least 85%, 90% and 95% of transcript length to other genes in the set (duplicate sequences were 100% similar). SnoRNA in equivalent sets are classified according to host gene biotype, designated antisense if on the opposite strand to an overlapping gene, else designated nongenic. **b** Observed proportion of genes in **a** at 85% similarity divided by the expected proportion. **c** Stacked bar chart of the numbers of snoRNA in **a** that are identifiable by a uniquely-mapping read. **d** Observed proportion of genes in **c** at 85% similarity divided by the expected proportion.

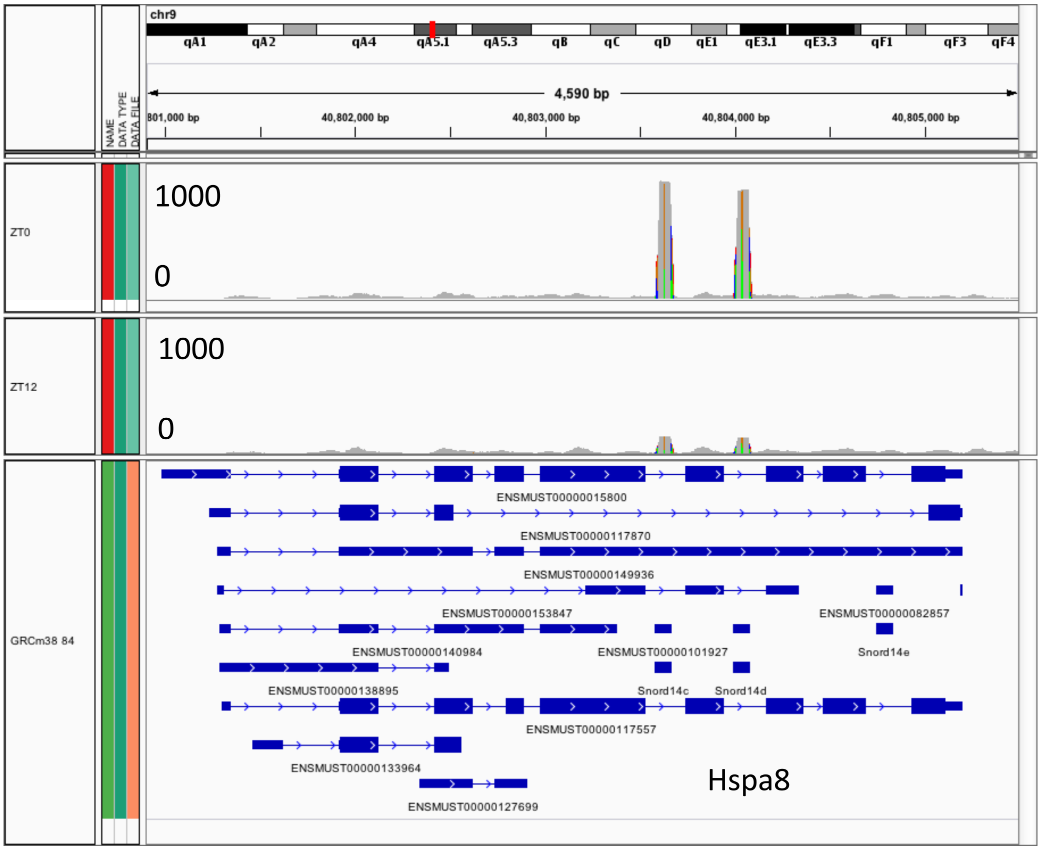

Figure S2: **Hspa8 locus.**
IVG browser view of the Hspa8 locus showing transcript models and snoRNAs Snord14c, Snord14d and Snord14e.

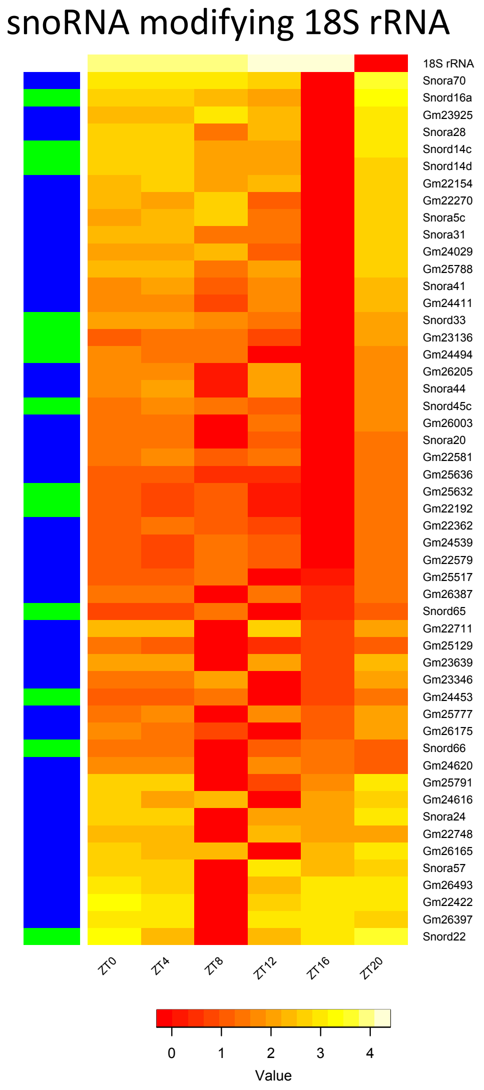

Figure S3: **SnoRNA modifying 18S and 28S rRNA.**

a Heatmap of the expression of 18S rRNA and snoRNA known to modify 18S. 18S rRNA (top row) has peak expression at ZT12-ZT16 whereas snoRNA known to interact with 18S have minimum expression at this time. Box H/ACA snoRNA are indicated by blue side colours and box C/D by green. **b** Fraction of snoRNA known to modify 28S whose expression is positively (solid red) and negatively (solid green) correlated with 28S expression at increasing values of R^2 (left). Similarly, for hosts of these snoRNA (right). The proportion of all genes with positive (dashed red) and negative (dashed green) correlation with 28S is shown for reference. Correlations were calculated for each gene after removing rRNA expression in each sample and rescaling to 10^6 to remove the effect of changes in rRNA on other genes across samples.

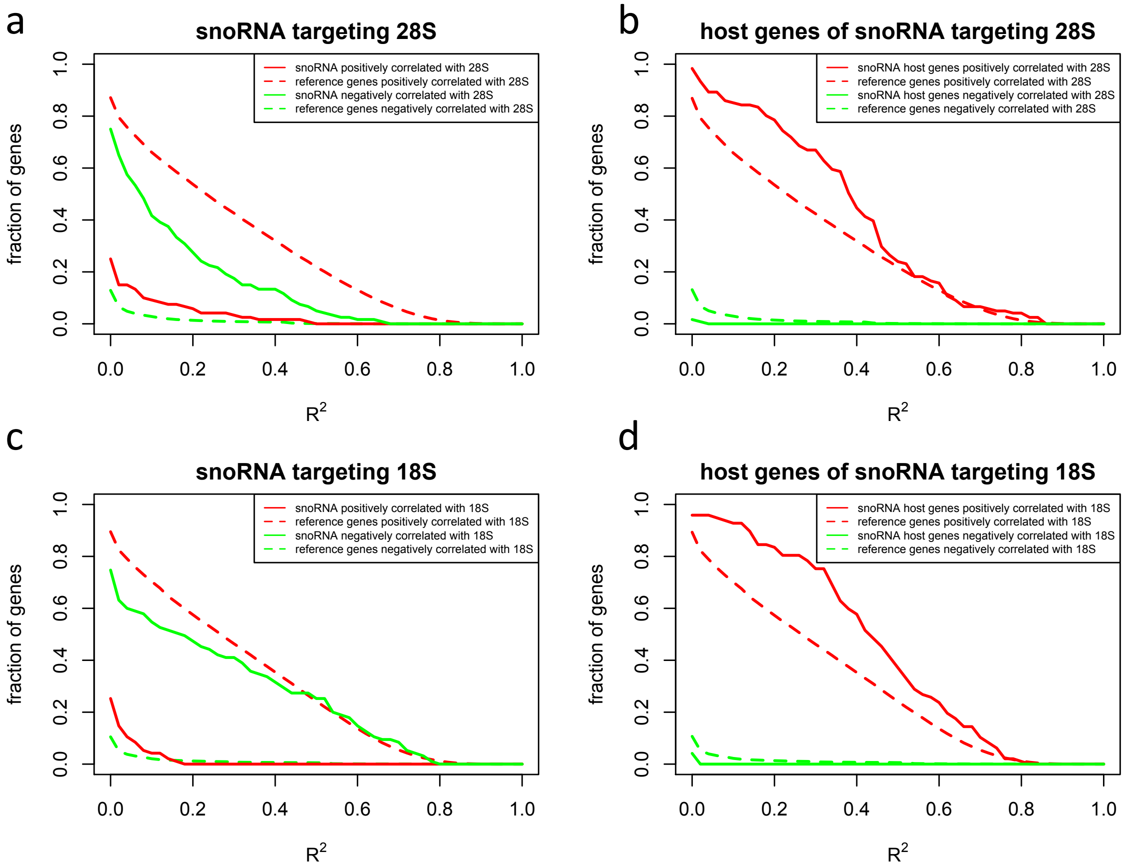

Figure S4: **Cumulative fractions of genes positively and negatively correlated with pre-rRNA expression.**
a The cumulative fractions of snoRNA known to modify 28S rRNA whose expression was positively (red solid line) and negatively (green solid line) correlated with 28S rRNA expression at R^2 values from 0 to 1. The fractions of a reference set of genes (all other genes) with positive and negative correlations is shown for comparison (red and green dashed lines). Fewer genes have significant correlations as the R^2 threshold increases. Negative correlations with 28S are rare in the reference genes (dashed green line) whereas for snoRNA they constitute the majority of significant correlations (solid green line) The cumulative fractions of snoRNA host genes are plotted similarly **b**, and the cumulative fractions of snoRNA modifying 18S and their hosts are are shown in **c** and **d**.

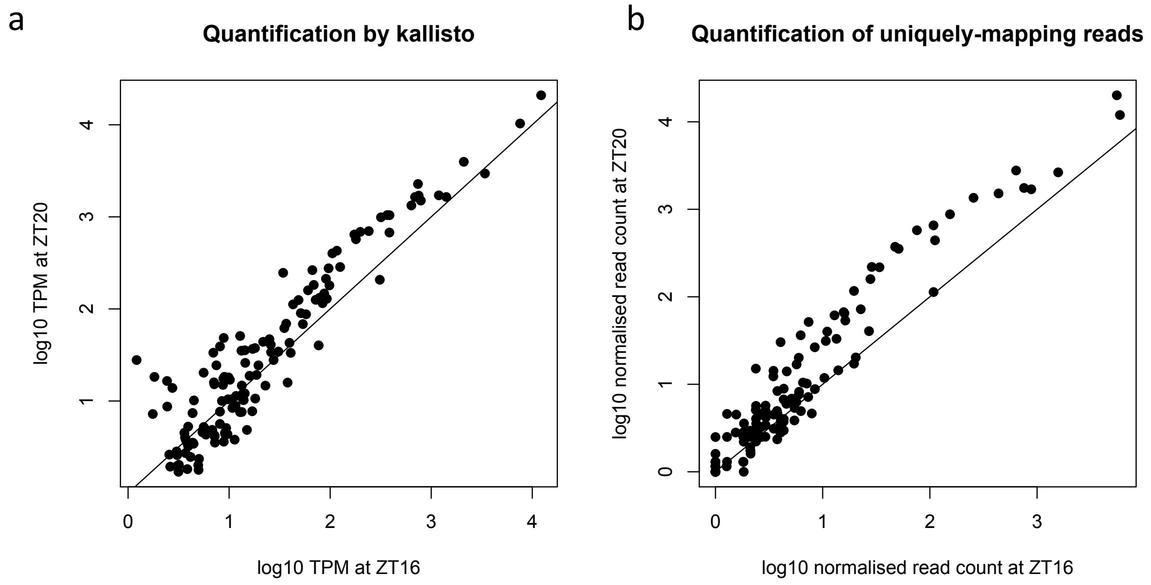

Figure S5: **Comparison of the expression of 28S-modifying snoRNA at ZT20 with expression at ZT16.**
a Scatterplot of snoRNA expression in TPM at ZT20 against expression at ZT16 quantified by Kallisto. **b** Scatterplot of snoRNA read counts at ZT20 against read counts at ZT16 quantified by uniquely-mapping reads (mapping by bowtie2, quantification by htseq-count and library depth normalised to 10^7). Plots shows all 28S-modifying snoRNA that could be found in the snOPY database.

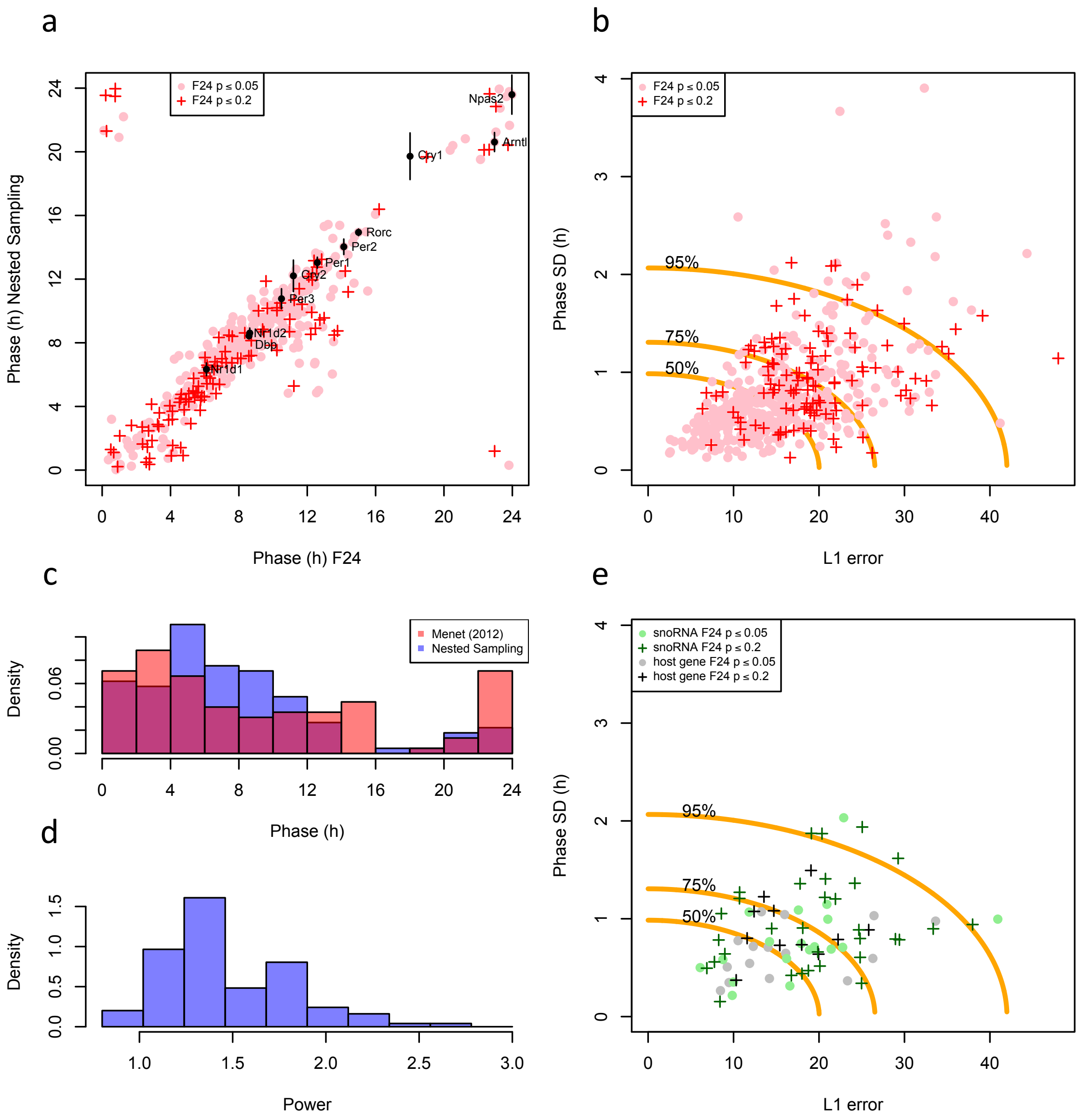

Figure S6: **Inference of circadian rhythms.**

a Scatterplot of phase computed by nested sampling against phase computed by the F24 method. Genes with FDR less than 0.2 are distinguished from those meeting a 0.05 threshold. Error bars computed by nested sampling (y axis) indicate one standard deviation. **b** Scatterplot of the standard deviation of cosine phase against the sum of residual error (L1 error) for the genes in **a** . Contours show constant values of the radial score that include increasing percentages of cyclic genes. **c** Histogram of phase of cyclic genes computed by nested sampling and values for these genes published by Menet et al. (2012) using alternative quantification and analysis. **d** Histogram of use of the power parameter (q). **e** Scatterplot of the standard deviation of cosine phase against the sum of residual error (L1 error) for snoRNA and host genes. Contours as in **b**.

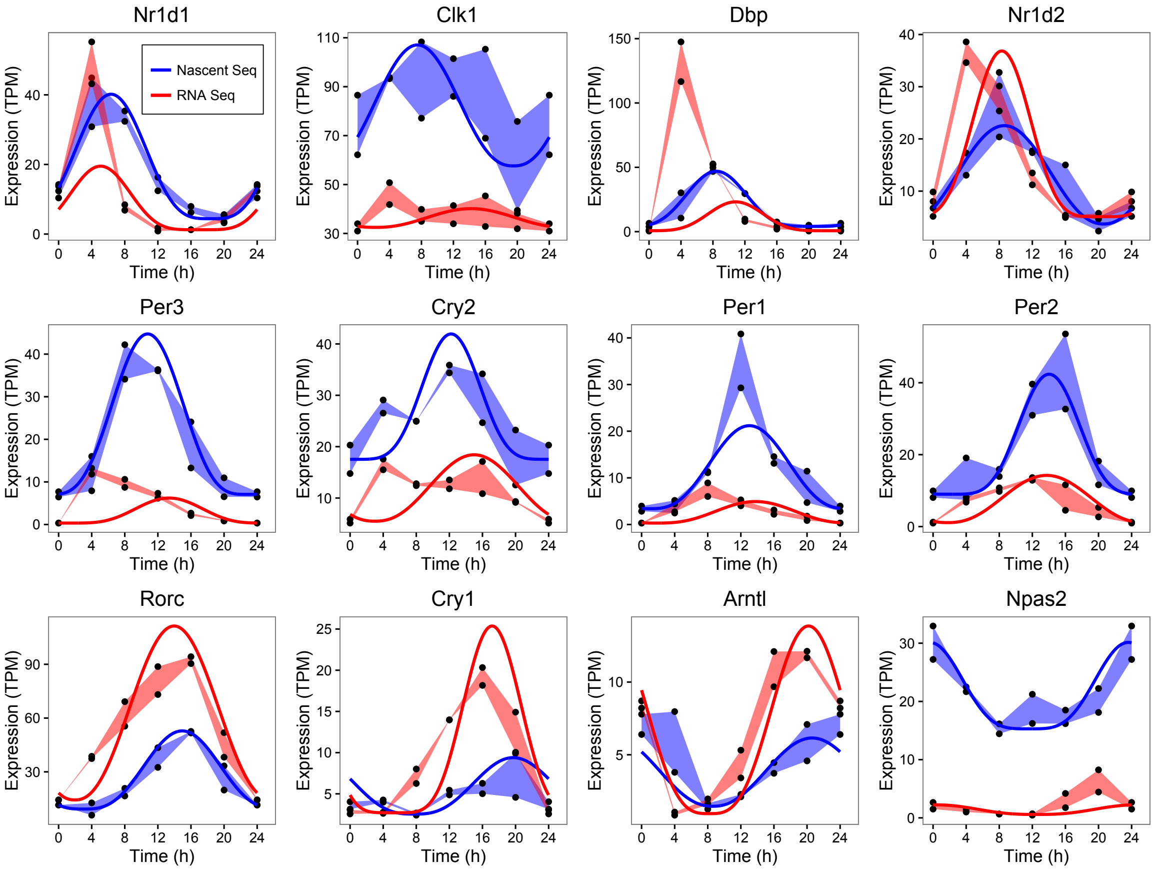

Figure S7: **Clock gene expression.**

Expression of twelve established clock genes plotted as a polygons between the maximum replicate data values across the time series and their minimum. Blue shaded areas represent nascent sequencing data, red represents RNA sequencing, and black symbols are data values. The solid lines are the best fit of the cosine^q model to the median of the replicates at each time point (blue nascent seq; red RNA seq).

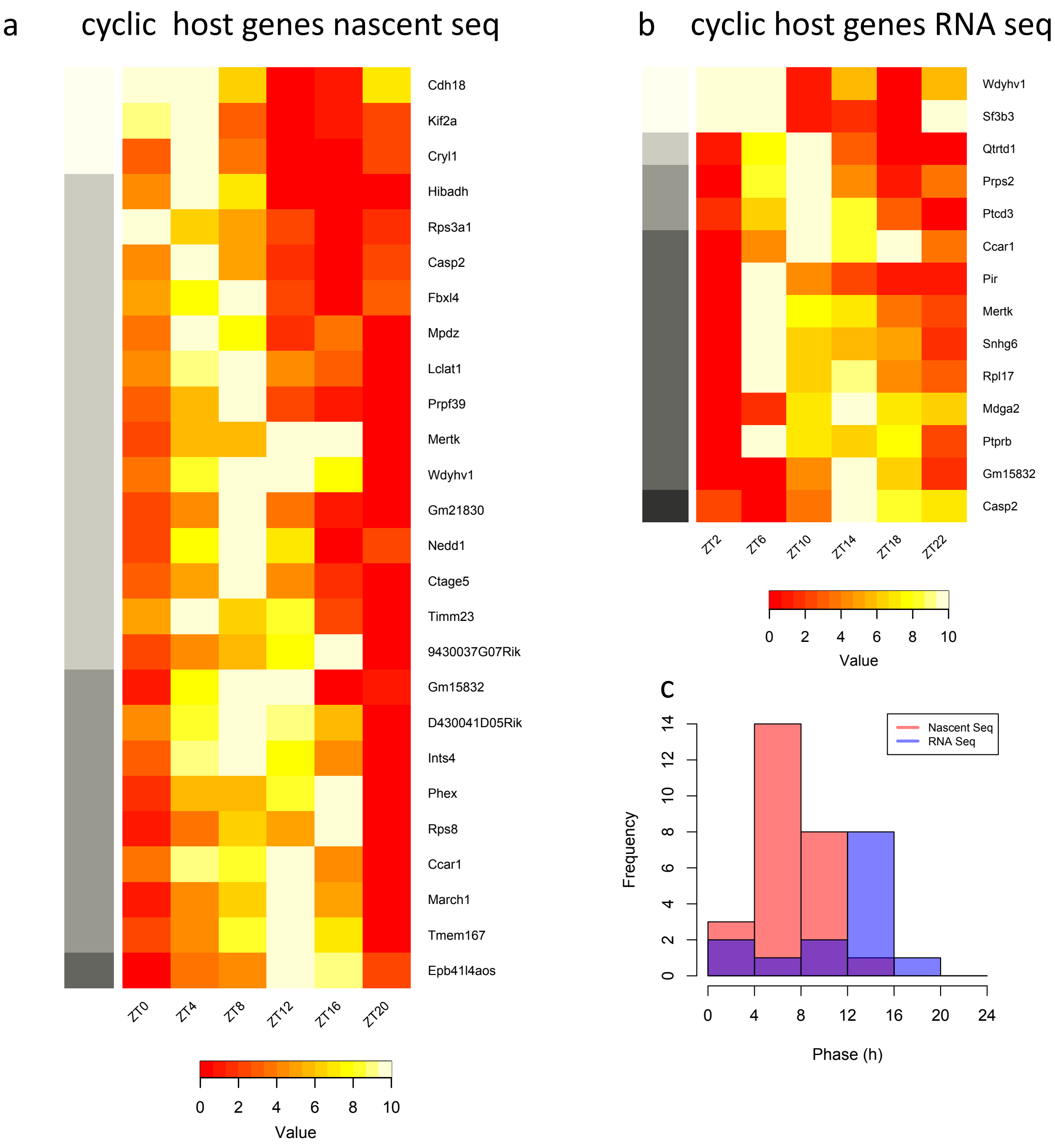

Figure S8: **Cyclically expressed snoRNA host genes.**
a Heatmap of the expression of snoRNAs host genes that are inferred to be cyclic in nascent sequencing. **b** Heatmap of the expression of snoRNAs host genes that are inferred to be cyclic in RNA sequencing. Row side colours (white-black) represent time of peak expression (phase). **c** Histogram of the phase of host genes in **a** and in **b**.

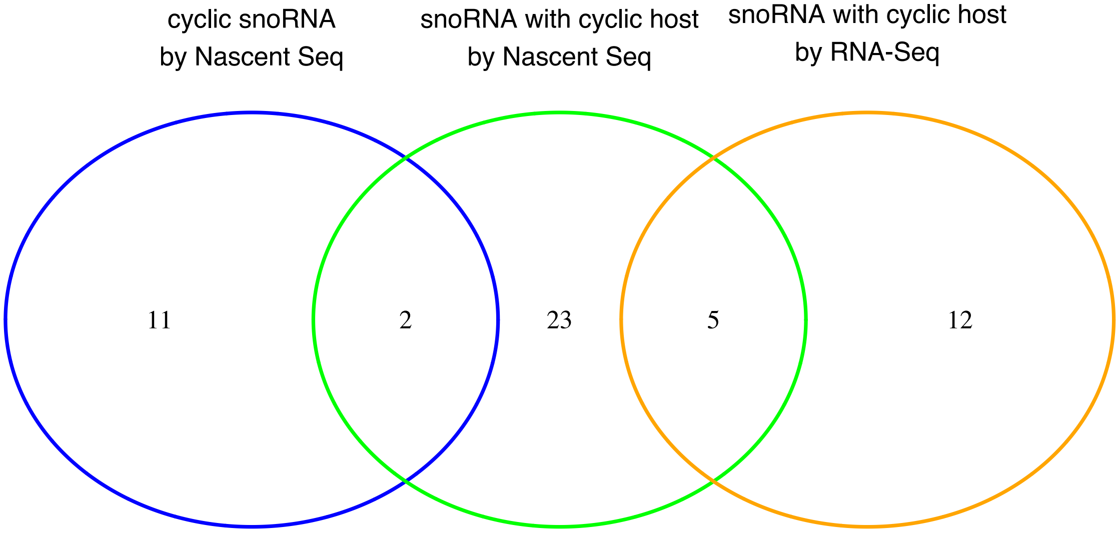

Figure S9: **Intersection between subsets of cyclic snoRNA.**
Venn diagram shows the numbers of snoRNA inferred to be cyclic and the number having a cyclic host, both by nascent sequencing and by RNA sequencing, and their intersections.

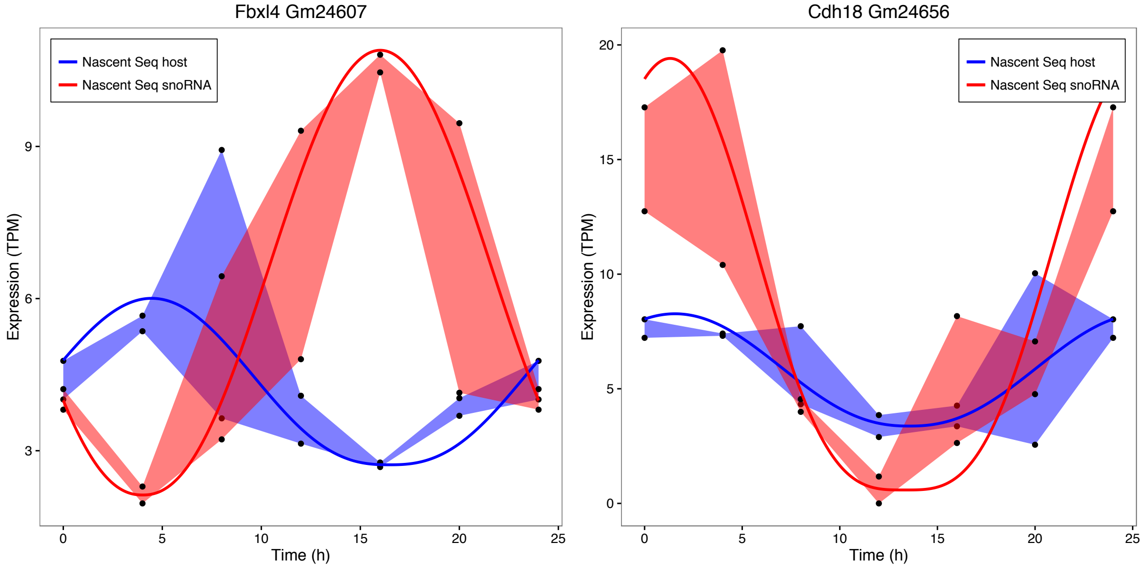

Figure S10: **SnoRNA and host gene expression.**
Nascent sequencing expression of the two cyclic SNORA17 genes (Gm24607 and Gm24656) with cyclic hosts (identified in Fig. S9) plotted as polygons together with their host gene expression (Fbxl4 and Cdh18 respectively).

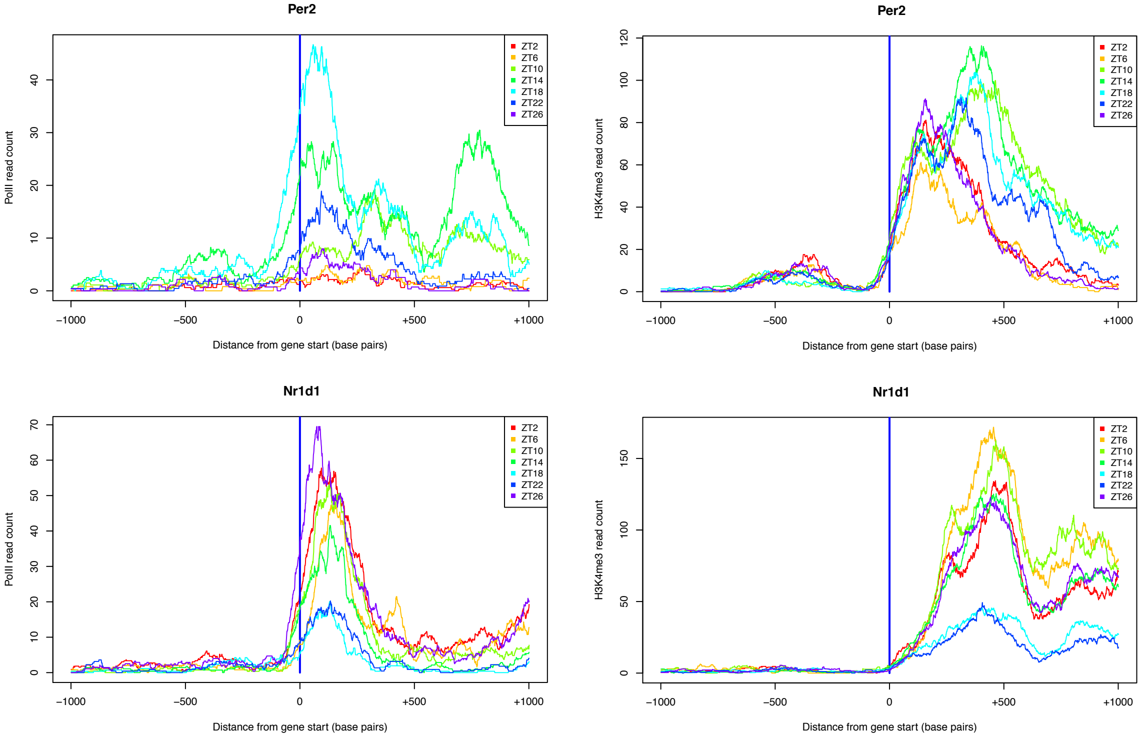

Figure S11: **PolII and H3K4me3 signals around clock genes.**
Normalised read depth in a 2kb region centered on the gene start, and oriented in the direction of transcription, is shown for clock genes Per2 and Nr1d1 for RNA Polymerase II (left) and for H3K4me3 ChIP sequencing data (right) data at seven time points.

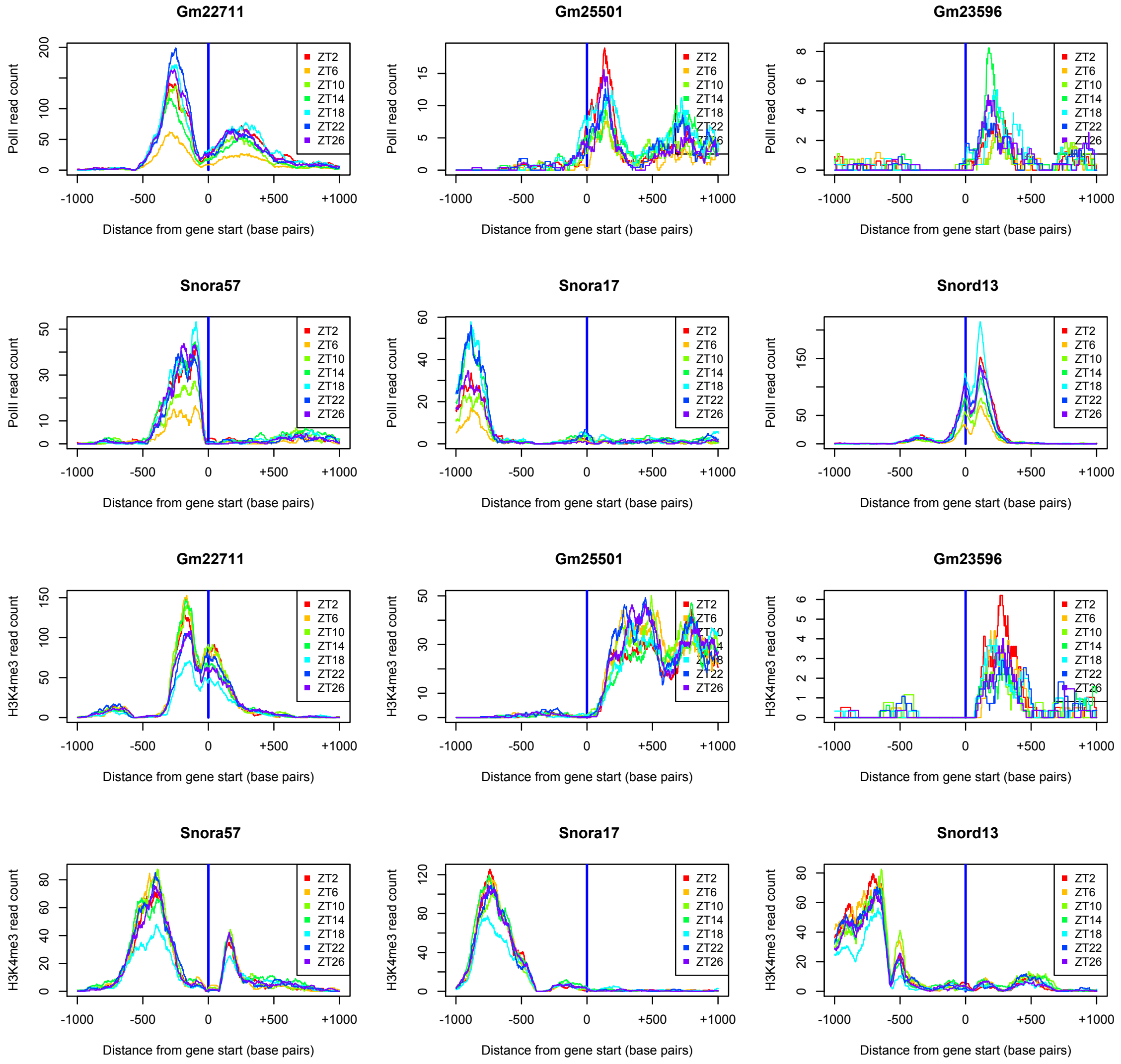

Figure S12: **PolII and H3K4me3 signals around independently transcribed snoRNA.** Normalised read depth in a 2kb region centered on the snoRNA gene start, and oriented in the direction of transcription, is shown for selected independently transcribed snoRNA for RNA Polymerase II (first and second rows) and for H3K4me3 ChIP sequencing data (third and fourth rows) data at seven time points. A peak in PolII over the gene and an adjacent peak in H3K4me3 are characteristic chromatin features. Note that Snora17 has a host gene in Refseq that is not annotated in Ensembl.

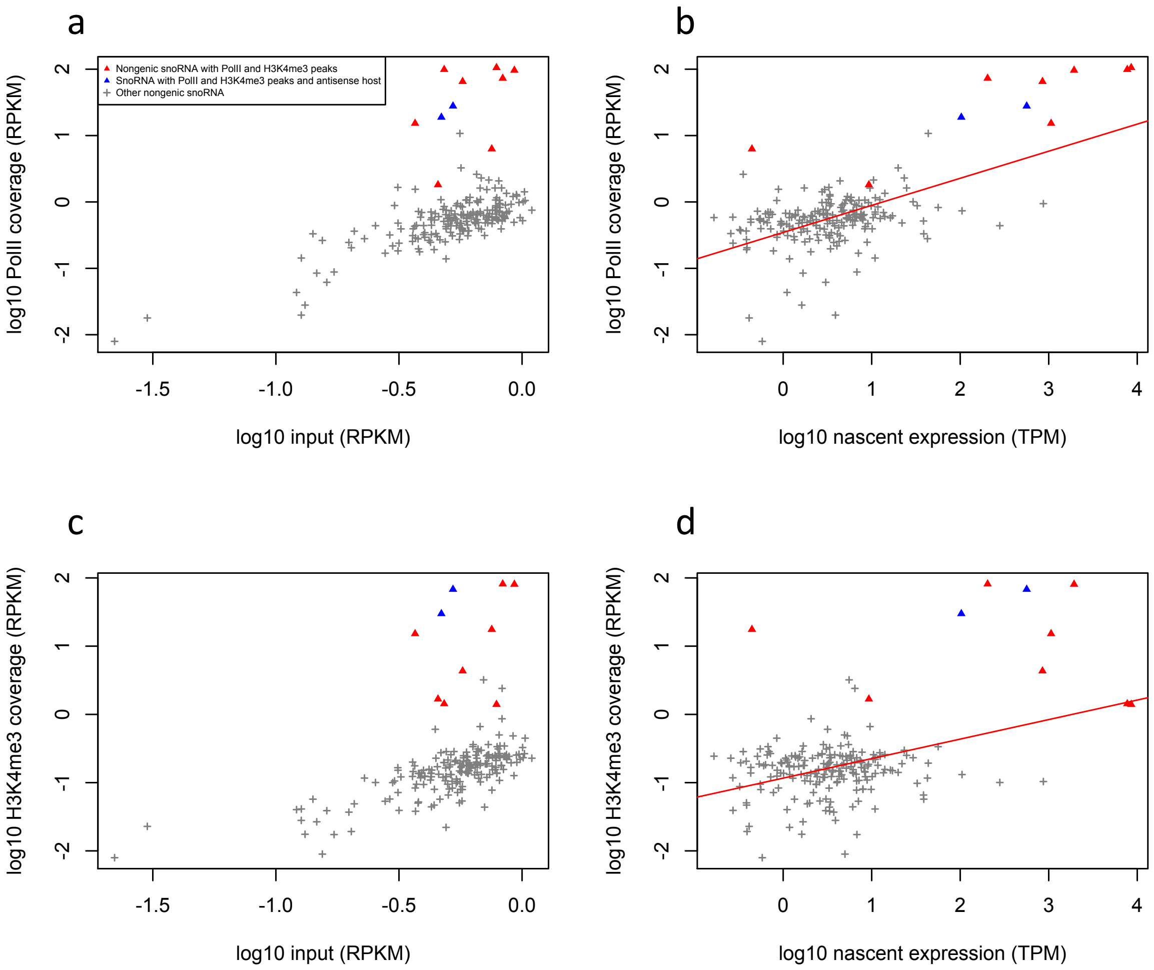

Figure S13: **Correlations between chromatin signals and gene expression.** **a** Scatterplot of mean RNA Polymerase II signal against mean input for nongenic snoRNA (coverage over gene expressed in RPKM, mean RPKM calculated over time course), distinguishing snoRNA overlapping peaks in PolII and H3K4me3 (red). **b** Scatterplot of mean RNA Polymerase II signal (RPKM) against mean nascent expression (mean TPM calculated over time course), red line shows regression (R^2=0.35, p≤2e-16). **c** Scatterplot of mean H3K4me3 signal against mean input for nongenic snoRNA (coverage over gene expressed in RPKM, mean RPKM calculated over time course). **d** Scatterplot of mean H3K4me3 signal (RPKM) against mean nascent expression (TPM), red line shows regression (R^2=0.17, p=6e-10). SnoRNA genes were extended by 200 bases upstream and downstream to capture immediately adjacent chromatin signals.

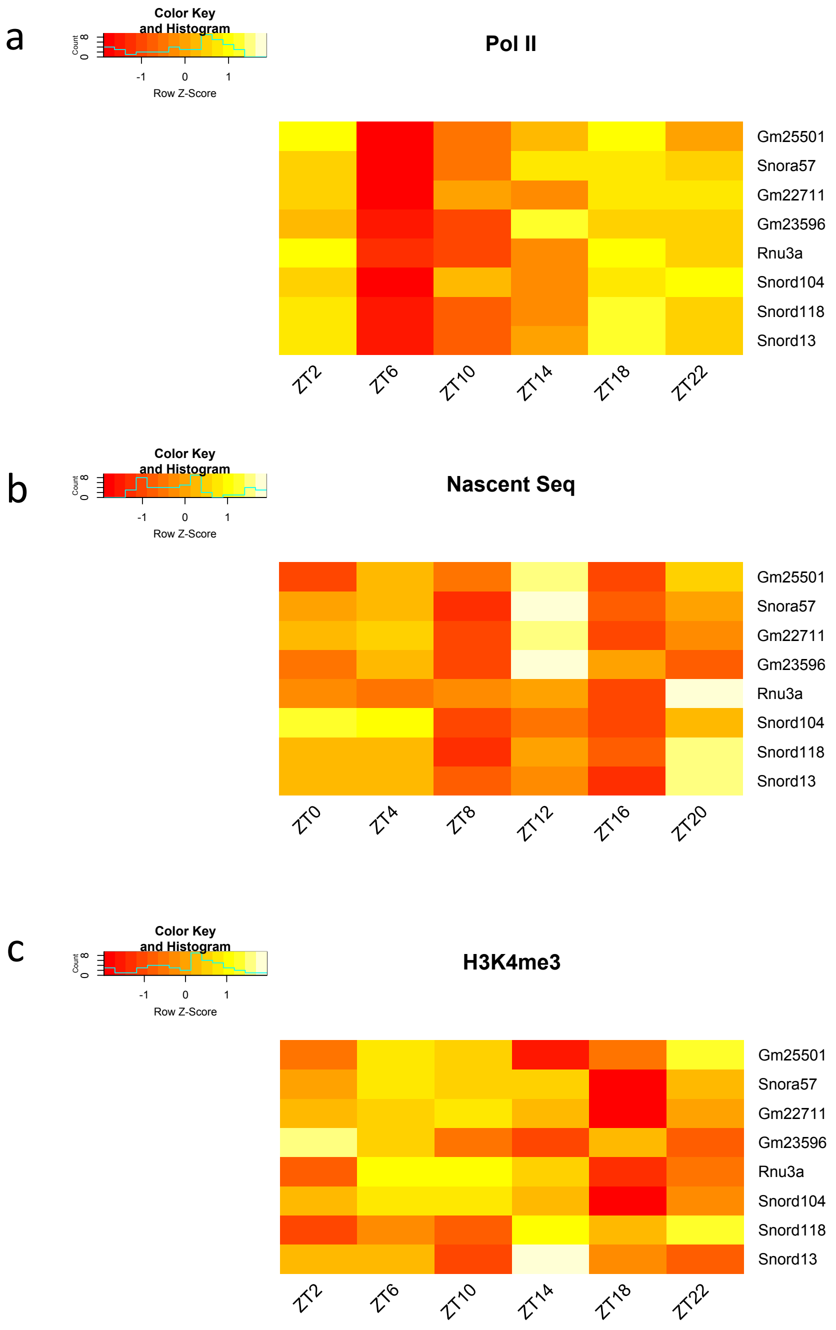

Figure S14: **Chromatin signals and gene expression of independently transcribed snoRNA over time.**
a Heatmap of log2 fold change in RNA Polymerase II signal compared with input over time for eight independently transcribed snoRNA. **b** Heatmap of nascent sequencing expression for snoRNA in **a**. **c** Heatmap of log2 fold change in H3K4ME3 signal compared with input over time for snoRNA in **a**.

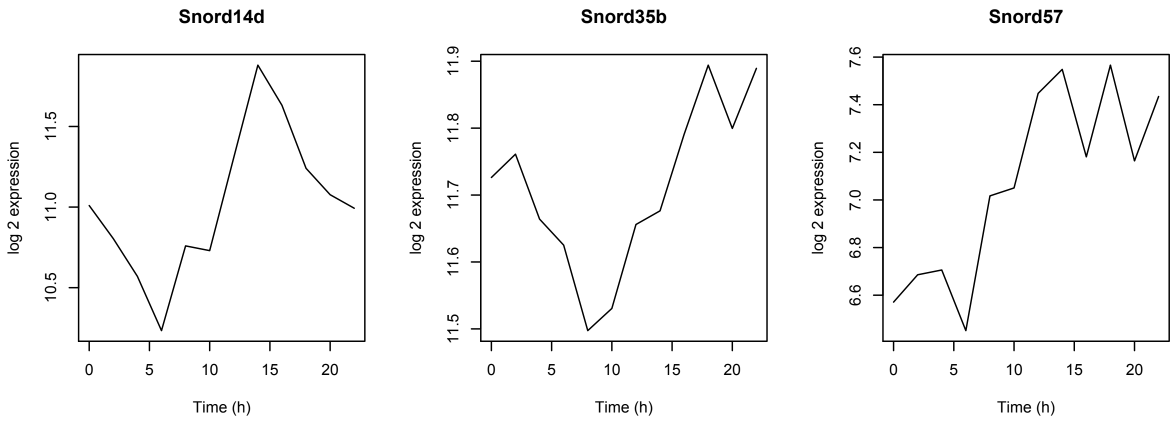

Figure S15: **SnoRNA expression from the circadian study of Jouffe et al. (2012).** Log2 expression over time of three snoRNA we found to be cyclic. Data is from GSE33726 and snoRNA probe mappings were taken from Ensembl GRCm38.
